# Supplementary material for: Mental health and health-related quality of life among adults with osteoarthritis: A national population-based study
Source: PLoS One. 2025 Jun 18;20(6):e0325780. doi: 10.1371/journal.pone.0325780 (PMC12176145; doi:10.1371/journal.pone.0325780)
Supplement: Attachment — Submitted filename: PONE-D-24-50708_R2.pdf [file pone.0325780.s003.pdf]

PLOS ONE

Mental Health and Health-Related Quality of Life among Adults with Osteoarthritis: A National Population-Based Study

--Manuscript Draft--

**Manuscript Number:**	PONE-D-24-50708R1
**Article Type:**	Research Article
**Full Title:**	Mental Health and Health-Related Quality of Life among Adults with Osteoarthritis: A National Population-Based Study
**Short Title:**	Depression and Anxiety and Quality of Life among Osteoarthritis
**Corresponding Author:**	Monira Alwhaibi<br>King Saud University<br>Riyadh, SAUDI ARABIA
**Keywords:**	Anxiety; Osteoarthritis; Depression; Quality of Life; Rand 12
**Abstract:**	Background: Adults with osteoarthritis are vulnerable to mental health disorders, which may have a significant impact on their Health-Related Quality of Life (HRQoL). Thus, the purpose of this study was to look at the relationship between anxiety and depression and HRQoL in adults with osteoarthritis.<br>Methods: Adult patients diagnosed with osteoarthritis who were 18 years of age or older were identified using data from the Medical Expenditure Panel Survey for the years 2018 through 2021. HRQoL was computed using the veterans RAND 12 item health survey which has two domains the Physical and Mental Component Summary (PCS & MCS) ratings. The link between anxiety and depression and HRQoL in individuals with osteoarthritis was examined using multivariable linear regression after the adjustment for a variety of covariates. Results: Of the 3,658 individuals with osteoarthritis that were identified, 12.0% experienced depression, 12.5% had anxiety, and 9.9% had both illnesses. The PCS and MCS mean scores of the HRQoL were lowest for persons with comorbid depression and anxiety compared to adults with osteoarthritis only. Moreover, from the adjusted regression analysis, adults with osteoarthritis who had depression (MCS: adjusted β = -5.408, p<0.001), anxiety (MCS: adjusted β = -3.485, p<0.001), or both depression and anxiety (MCS: adjusted β = -10.348, p-value<0.0001) had significantly lower HRQoL MCS scores compared to those with osteoarthritis only. Those who were physically active and employed had notably better scores on the PCS and MCS than those who were not.<br>Conclusions: This nationally representative sample has shed light on the relationships between anxiety, depression, and a low HRQoL in adults with osteoarthritis. The study also revealed the impact of socio-economic factors such as education level and income on HRQoL, and the benefits of employment and regular exercise. Importantly, the findings underscore the need for lifestyle modifications to reduce anxiety and depression in individuals with osteoarthritis. They also highlight the practical implications for healthcare planning and resource allocation, providing valuable insights for policymakers and healthcare professionals.
**Order of Authors:**	Monira Alwhaibi
	Tariq M. Alhawassi
**Response to Reviewers:**	Response to Reviewer Comments<br>We appreciate the reviewer’s thoughtful comments and suggestions, which have helped improve the clarity and quality of our manuscript. Below is our detailed response to each comment:<br>Data Analysis:<br>Comment 1: Inclusion of Candidate Variables in Multivariable Linear Regression Model<br>Response: Thank you for this comment; we have now clarified the selection process of variables in the Methods section. Variables were included based on their theoretical relevance, clinical importance, and prior literature supporting their association with health-related quality of life (HRQoL) in osteoarthritis (OA) patients. Additionally, a bivariate analysis was performed, and only variables with a p-value <0.05 were considered for the multivariable regression model.<br>Results:

Powered by Editorial Manager® and ProduXion Manager® from Aries Systems Corporation

	Comment 2: Likelihood Ratio p-value Indication<br>Response: We have ensured that the reported p-values are based on the likelihood ratio test where appropriate and have explicitly indicated this in the text and tables.<br>Comment 3: Reporting All Significant Results<br>Response: We have revised the results section to ensure that all significant findings are reported.<br>Comment 4: Specificity about Domains Being Reported<br>Response: Thanks for pointing out, now we have specify whether the reported HRQoL results pertain to the Physical Component Summary (PCS) or the Mental Component Summary (MCS) to enhance clarity.<br>Comment 5: Comparison with the Reference Group<br>Response: Thanks for this comment; we have ensured that all the reported regression results include a clear comparison with the reference group.<br><br><br>Discussion:<br>Comment 6: Addressing Significant vs. Insignificant Results<br>Response: The discussion has been revised to emphasize significant results while removing discussion on insignificant findings (i.e., gender was previously discussed despite being statistically insignificant; this has now been removed).<br>Comment 7: Comprehensive Reporting of Key Variables<br>Response: As per the reviewer recommendations, we have expanded the discussion to cover all significant factors, including perceived health, employment, region, and income, as they were significantly associated with HRQoL. We have also clarified the role of education by providing possible explanations for the observed findings and comparing them with previous studies.<br>Recommendations:<br>Comment 8: Specificity in Recommendations<br>Response: Thanks for the suggestion. Recommendations have been revised to clearly address specific entities such as healthcare providers, policymakers, and public health organizations.<br>Comment 9: Re-editing and Completing References<br>Response: The reference section has been thoroughly revised to ensure completeness, correct formatting, and accuracy.
**Additional Information:**	
**Question**	**Response**
**Financial Disclosure**<br><br>Enter a financial disclosure statement that describes the sources of funding for the work included in this submission. Review the submission guidelines for detailed requirements. View published research articles from *PLOS ONE* for specific examples.<br><br>This statement is required for submission and **will appear in the published article** if the submission is accepted. Please make sure it is accurate.	The author(s) received no specific funding for this work.

Powered by Editorial Manager® and ProduXion Manager® from Aries Systems Corporation

**Funded studies**<br>Enter a statement with the following details:<br>• Initials of the authors who received each award<br>• Grant numbers awarded to each author<br>• The full name of each funder<br>• URL of each funder website<br>• Did the sponsors or funders play any role in the study design, data collection and analysis, decision to publish, or preparation of the manuscript?<br><br>Did you receive funding for this work?	
**Competing Interests**<br><br>Use the instructions below to enter a competing interest statement for this submission. On behalf of all authors, disclose any competing interests that could be perceived to bias this work—acknowledging all financial support and any other relevant financial or non-financial competing interests.<br><br>This statement is required for submission and **will appear in the published article** if the submission is accepted. Please make sure it is accurate and that any funding sources listed in your Funding Information later in the submission form are also declared in your Financial Disclosure statement.<br><br>View published research articles from *PLOS ONE* for specific examples.	The authors have declared that no competing interests exist

**NO authors have competing interests**<br><br>Enter: *The authors have declared that no competing interests exist.*<br><br>**Authors with competing interests**<br><br>Enter competing interest details beginning with this statement:<br><br>*I have read the journal's policy and the authors of this manuscript have the following competing interests: [insert competing interests here]*<br><br>* typeset	
**Ethics Statement**<br><br>Enter an ethics statement for this submission. This statement is required if the study involved:<br><br>• Human participants<br>• Human specimens or tissue<br>• Vertebrate animals or cephalopods<br>• Vertebrate embryos or tissues<br>• Field research<br><br>Write "N/A" if the submission does not require an ethics statement.<br><br>General guidance is provided below. Consult the submission guidelines for detailed instructions. **Make sure that all information entered here is included in the Methods section of the manuscript.**	No ethical review or approval was needed as we have utilized a publicly accessible, the Medical Expenditure Panel Survey (MEPS) database.

Powered by Editorial Manager® and ProduXion Manager® from Aries Systems Corporation

**Format for specific study types**<br><br>**Human Subject Research (involving human participants and/or tissue)**<br>• Give the name of the institutional review board or ethics committee that approved the study<br>• Include the approval number and/or a statement indicating approval of this research<br>• Indicate the form of consent obtained (written/oral) or the reason that consent was not obtained (e.g. the data were analyzed anonymously)<br><br>**Animal Research (involving vertebrate animals, embryos or tissues)**<br>• Provide the name of the Institutional Animal Care and Use Committee (IACUC) or other relevant ethics board that reviewed the study protocol, and indicate whether they approved this research or granted a formal waiver of ethical approval<br>• Include an approval number if one was obtained<br>• If the study involved *non-human primates*, add *additional details* about animal welfare and steps taken to ameliorate suffering<br>• If anesthesia, euthanasia, or any kind of animal sacrifice is part of the study, include briefly which substances and/or methods were applied<br><br>**Field Research**<br><br>Include the following details if this study involves the collection of plant, animal, or other materials from a natural setting:<br>• Field permit number<br>• Name of the institution or relevant body that granted permission	
**Data Availability**<br><br>Authors are required to make all data underlying the findings described fully available, without restriction, and from the time of publication. PLOS allows rare exceptions to address legal and ethical concerns. See the PLOS Data Policy and FAQ for detailed information.	Yes - all data are fully available without restriction

Powered by Editorial Manager® and ProduXion Manager® from Aries Systems Corporation

A Data Availability Statement describing where the data can be found is required at submission. Your answers to this question constitute the Data Availability Statement and **will be published in the article**, if accepted.<br><br>**Important:** Stating 'data available on request from the author' is not sufficient. If your data are only available upon request, select 'No' for the first question and explain your exceptional situation in the text box.<br><br>Do the authors confirm that all data underlying the findings described in their manuscript are fully available without restriction?	
**Describe where the data may be found in full sentences. If you are copying our sample text, replace any instances of XXX with the appropriate details.**<br><br>• If the data are **held or will be held in a public repository**, include URLs, accession numbers or DOIs. If this information will only be available after acceptance, indicate this by ticking the box below. For example: *All XXX files are available from the XXX database (accession number(s) XXX, XXX.).*<br>• If the data are all contained **within the manuscript and/or Supporting Information files**, enter the following: *All relevant data are within the manuscript and its Supporting Information files.*<br>• If neither of these applies but you are able to provide **details of access elsewhere**, with or without limitations, please do so. For example:<br><br>*Data cannot be shared publicly because of [XXX]. Data are available from the XXX Institutional Data Access / Ethics Committee (contact via XXX) for researchers who meet the criteria for access to confidential data.*<br><br>*The data underlying the results presented in the study are available from (include the name of the third party*	The dataset used in this study is available from the MEPS database at this URL: https://meps.ahrq.gov/data_stats.

and contact information or URL).<br>• This text is appropriate if the data are owned by a third party and authors do not have permission to share the data.<br><br>* typeset	
Additional data availability information:	

Powered by Editorial Manager® and ProduXion Manager® from Aries Systems Corporation

Manuscript

Click here to access/download;Manuscript;Osteoarthrities_HRQoL_05-03-202

Mental Health and Health-Related Quality of Life among Adults with Osteoarthritis: A National Population-Based Study

Running Title: Depression and Anxiety and Quality of Life among Osteoarthritis

Monira Alwhaibi[1,2*¶] Tariq M. Alhawassi[1,2¶]

[1]Department of Clinical Pharmacy, College of Pharmacy, King Saud University, Riyadh, Saudi Arabia
[2] Medication Safety Research Chair, College of Pharmacy, King Saud University, Riyadh, Saudi Arabia

¶These authors contributed equally to this work.

*Corresponding author: Monira Alwhaibi, MS, PhD, Professor, Department of Clinical Pharmacy, King Saud University, College of Pharmacy, Riyadh, 11149, Saudi Arabia, E-mail: malwhaibi@ksu.edu.sa

ORCID ID Corresponding Author: 0000-0003-3707-0582

Abstract

Background: Adults with osteoarthritis are vulnerable to mental health disorders, which may have a significant impact on their Health-Related Quality of Life (HRQoL). Thus, the purpose of this study was to look at the relationship between anxiety and depression and HRQoL in adults with osteoarthritis.

Methods: Adult patients diagnosed with osteoarthritis who were 18 years of age or older were identified using data from the Medical Expenditure Panel Survey for the years 2018 through 2021. HRQoL was computed using the veteran's RAND 12-item health survey which has two domains the Physical and Mental Component Summary (PCS & MCS) ratings. The link between anxiety and depression and HRQoL in individuals with osteoarthritis was examined using multivariable linear regression after the adjustment for a variety of covariates. **Results:** Of the 3,658 individuals with osteoarthritis that were identified, 12.0% experienced depression, 12.5% had anxiety, and 9.9% had both illnesses. The PCS and MCS mean scores of the HRQoL were lowest for persons with comorbid depression and anxiety compared to adults with osteoarthritis only. Moreover, from the adjusted regression analysis, adults with osteoarthritis who had depression (MCS: adjusted $\beta = -5.408$, $p<0.001$), anxiety (MCS: adjusted $\beta = -3.485$, $p<0.001$), or both depression and anxiety (MCS: adjusted $\beta = -10.348$, p-value<0.0001) had significantly lower HRQoL MCS scores compared to those with osteoarthritis only. Those who were physically active and employed had notably better scores on the PCS and MCS than those who were not.

Conclusions: This nationally representative sample has shed light on the relationships between anxiety, depression, and a low HRQoL in adults with osteoarthritis. The study also revealed the impact of socioeconomic factors such as education level and income on HRQoL, and the benefits of employment and regular exercise. Importantly, the findings underscore the need for lifestyle modifications to reduce anxiety and depression in individuals with osteoarthritis. They also highlight the practical implications for healthcare planning and resource allocation, providing valuable insights for policymakers and healthcare professionals.

Keywords. Anxiety; Osteoarthritis; Depression; Quality of Life; Rand 12.

1. Introduction

Osteoarthritis (OA) is one of the most prevalent disabling illnesses globally and has been shown to have serious adverse effects on health [1]. It was estimated that OA affected around six hundred million individuals worldwide in 2020, representing 7.6% of the world's population [2]. Moreover, OA cases are expected to rise by nearly 50% for the hand, 75% for the knee and the hip, and up to 95% increase in OA prevalence for other types by 2050 as compared to 2020 [2]. Nowadays, OA is the 14th leading cause of age-standardized years lived with disability (YLDs) globally and has a substantial burden on individuals characterized by pain, limitations of activity, and diminished quality of life [2]. In addition to the direct health-related burden, OA has a significant financial burden and detrimental effects on a person's psychological health and well-being, work participation, sleep, mobility, and diminished health-related quality of life (HRQoL) [1, 3-10]. Therefore, individuals with OA have three times more potential than individuals in the general population to experience anxiety and depression, which could be attributed to pain severity, poor function, number of OA sites, immunological inflammation, and structural alterations in the brain [11-13].

Anxiety and depression can increase the risk of adverse clinical outcomes, such as OA illness burden and morbidity, and also result in increased healthcare costs and health service utilization, with higher healthcare provider visits, emergency room visits, and hospitalizations [12, 14-16]. Besides, depression was associated with lower HRQoL for individuals with OA [17, 18]. HRQoL is a crucial outcome measure for evaluating the overall effects of long-term illnesses like OA, including a person's social, emotional, and physical well-being [19].

With the aforementioned risk of OA on patients' psychological well-being, maintaining a high quality of life in terms of health despite the difficulties posed by OA is a primary objective

for adult OA sufferers and their healthcare providers. Therefore, understanding the causes underlying a lower HRQoL becomes essential, especially when considering mental health. Yet, the relationship between depression and anxiety and the HRQoL of people with OA is not fully understood, given that the number of studies that evaluated this relationship is limited either in terms of generalizability (most of the published research was conducted in Japan and Korea) or limited in terms of methods used in identifying associations [6, 12, 15, 17-21]. Consequently, to optimize OA patient health, healthcare providers must have a thorough understanding of the mutual interaction that exists between depression, anxiety, and the general impact on HRQoL among such vulnerable populations. Therefore, this led us to conduct this research, which aimed at evaluating the association between depression, anxiety, and the HRQoL among adults with OA using a sample from a representative United States (US) database and adding more insightful knowledge to the currently available research in this significant field.

2. Methods

2.1. Study Design and Data

This study used Data from the Medical Expenditure Panel Survey (MEPS) from 2018 to 2021 to conduct a cross-sectional study. MEPS is a nationwide survey that gathers information on sociodemographics, health insurance, medical issues, medication use, and medical care from United States (US) citizens.

2.2. Study Population

According to the study's inclusion criteria, an adult must be between 18 and 64 years old, have a diagnosis of OA, alive within the study period, and have no missing data on HRQoL. The International Classification of Diseases, tenth Revision, Clinical Modification (ICD-10-CM)

clinical diagnosis codes were used to identify OA from MEPS data (M16: osteoarthritis of the hip, M17: osteoarthritis of the knee, M19: other and unspecified osteoarthritis) [22].

2.3. Measures

2.3.1. Outcome: Health-Related Quality of Life (HRQoL)

To evaluate the HRQoL, adults who participated in the MEPS filled out a questionnaire using a reliable and valid instrument, the Veterans RAND 12 Item Health Survey (VR-12©) [23-25]. VR-12 is a 12-item patient-reported outcome measure that evaluates eight domains of health: general health, physical functioning, role physical, bodily pain, vitality, role emotional, mental health, and social functioning [23]. The VR-12 has two parts: 1/ Physical Component Summary (PCS), which included responses from the general health, physical functioning, role physical, and bodily pain domains, and 2/ Mental Component Summary (MCS), which included responses from role emotional, vitality, mental health, and social functioning domains [26]. Scores range from 0 to 100, with higher PCS and MCS scores as better physical and mental HRQoL indicators.

2.4.2. Independent Variables

The primary independent variable of interest in this study was the different OA groups, each of which was mutually exclusive (OA only, OA and anxiety, OA and depression, and OA and both mental illnesses). Other independent variables were selected based on their documented influence on HRQoL in adults, both in general and those with OA, as reported in the literature including the social determinants (e.g., age, gender, education, occupational status, household income), chronic illnesses, physical activity, health insurance coverage, and perceived health [27-33].

2.5. Statistical Analyses

Descriptive statistics were employed to show the study sample's baseline characteristics, including mean, standard deviation, frequencies, and percentages. The differences in individual characteristics between the OA groups were evaluated using chi-square tests. The mean variances in HRQoL by OA groups were determined using ANOVA followed by Tukey's post hoc test for multiple comparisons to identify which specific groups differed. To assess the link between OA groups and HRQoL, we used multivariable linear regression and adjusted for all independent covariates. Variables were included in the model based on their theoretical relevance, clinical importance, and prior literature supporting their association with health-related quality of life (HRQoL) in OA patients [27-33]. Additionally, a bivariate analysis was performed and only variables with a likelihood ratio p-value of <0.05 were considered for the multivariable regression model. All the underlying assumptions of the linear regression model have been evaluated to ensure that they are satisfied by the regression model and are met by the variables. Those assumptions include correct model specification, normality, homoscedasticity, no autocorrelation, and no multicollinearity. After checking the underlying assumptions, we performed the linear regression model. All estimations in the statistical analysis considered the complicated survey design of the MEPS by integrating variance adjustment weights (strata and primary sample unit) from the MEPS with person-level weights. The Statistical Analysis System, SAS 9.4 (SAS Institute Inc., Cary, NC, USA), was used to analyze the data.

3. Results

3.1. Characteristics of the Study Sample

Table 1 lists the characteristics of the study sample, 3,658 adults with OA; the majority of the study sample was women (62.3%), aged 50 to 64 (72.0%), White (73.3%), and had higher than

high school (87.8%). About 12.0% of adults with OA also experienced depression, 12.5% had anxiety, and 9.9% had both illnesses.

Women with OA showed significantly higher rates of depression (13.6% vs. 9.3%), anxiety (14.7% vs. 9.0%), and comorbid anxiety and depression (12.0% vs. 6.5%, p-value = <0.0001) when compared to men. Furthermore, unemployed OA patients had significantly higher rates of depression (16.2% vs. 9.3%) and comorbid anxiety and depression (13.7% vs. 7.5%, p-value <0.0001) than employed OA patients. OAs adults with diabetes, asthma, COPD, and GERD had significantly higher rates of comorbid depression and anxiety than those without these comorbidities (p-value <0.0001).

Table 1. Characteristics of the Study Sample, n=3,658 Adults with Osteoarthritis (OA), Number and Row % of Sample Characteristics by OA Groups

	Total Sample		OA Only		OA & Depression		OA & Anxiety		OA & Depression & Anxiety		
	**N**	**Wt.%**	**N**	**Wt.%**	**N**	**Wt.%**	**N**	**Wt.%**	**N**	**Wt.%**	**P-value***
**All**	3,658	100.0	2361	65.6	473	12.0	429	12.5	395	9.9	
**Age in years**											
22-39	327	9.7	187	54.4	31	10.8	52	16.8	57	18.0	<0.0001
40-49	648	18.2	384	62.4	87	12.7	90	13.4	87	11.5	
50-64	2683	72.0	1790	67.9	355	12.0	287	11.7	251	8.4	
**Gender**											
Women	2405	62.3	1437	59.8	336	13.6	319	14.7	313	12.0	<0.0001
Men	1253	37.7	924	75.2	137	9.3	110	9.0	82	6.5	
**Race/ethnicity**											
White	2304	73.3	1412	63.5	318	12.4	305	13.8	269	10.2	0.011
African American	658	12.5	482	73.6	66	9.5	62	8.5	48	8.4	
Latino	488	8.8	336	72.1	58	11.3	42	8.1	52	8.5	
Others	208	5.4	131	64.9	31	13.0	20	11.1	26	11.0	
**Marital status**											
Married	1749	54.8	1232	71.0	190	10.3	199	12.2	128	6.5	<0.0001
Widow/Sep/Div	1250	30.2	712	57.6	200	14.1	166	13.9	172	14.4	
Never married	659	15.0	417	62.1	83	13.7	64	11.0	95	13.2	
**Education level**											
< High School	197	3.4	133	70.8	24	12.2	22	9.8	18	7.2	0.015
High School	395	8.0	240	61.7	54	13.2	38	8.7	63	16.5	
> High School	3033	87.8	1965	65.7	392	11.9	365	13.0	311	9.4	

Commented [P1]: Revise this title; it seems not clear… maybe…. "Socio-economic and health-related characteristic of study participants across osteoarthritis groups" This might be modified as necessary…

**Also, check "Heart ???" I added the question mark to draw your attention to the hanging word…... "Heart" disease? Please, clarify.

**Region**											
Northeast	589	16.9	385	68.3	70	9.4	57	10.1	77	12.2	0.356
Mid-west	939	26.3	572	63.4	145	13.9	113	12.2	109	10.4	
South	1444	38.8	946	64.9	169	12.5	175	13.1	154	9.5	
West	686	18.0	458	67.7	89	10.5	84	14.1	55	7.7	
**Employment**											
Employed	1942	60.8	1399	72.0	192	9.3	198	11.3	153	7.5	<0.0001
Not employed	1716	39.2	962	55.7	281	16.2	231	14.4	242	13.7	
**Poverty status**											
Poor	814	15.8	441	50.7	127	15.8	114	15.5	132	18.0	<0.0001
Near Poor	723	16.0	423	57.7	112	16.8	93	13.3	95	12.2	
Middle Income	911	24.8	606	66.4	123	12.7	101	12.4	81	8.6	
High Income	1210	43.5	891	73.5	111	8.4	121	11.2	87	6.8	
**Health Insurance**											
Private	2098	67.0	1507	71.9	227	9.8	210	11.2	154	7.1	<0.0001
Public	1415	29.4	755	51.3	227	16.6	207	15.7	226	16.4	
Uninsured	145	3.6	99	65.5	19	14.0	12	11.2	15	9.3	
**Rx Insurance**											
Rx insurance	1850	59.4	1347	72.8	185	9.3	187	11.1	131	6.8	<0.0001
No Rx insurance	1808	40.6	1014	55.1	288	15.9	242	14.5	264	14.4	
**General health**											
Excellent/very good	1002	31.3	770	77.2	100	8.5	96	10.9	36	3.4	<0.0001
Good	1302	36.8	876	66.8	154	11.7	146	11.9	126	9.6	
Fair/poor	1354	31.9	715	52.8	219	15.8	187	14.8	233	16.6	
**Physical activity**											
3/week	1496	43.1	1054	71.2	169	9.8	156	12.0	117	7.0	<0.0001
No exercise	2158	56.8	1305	61.4	302	13.6	273	12.9	278	12.1	
**Heart???**											
Yes	422	10.5	235	58.6	63	14.7	62	13.8	62	12.8	0.111
No	3236	89.5	2126	66.4	410	11.7	367	12.4	333	9.5	
**Hypertension**											
Yes	1820	45.9	1107	62.6	272	14.2	236	12.9	205	10.2	0.017
No	1838	54.1	1254	68.2	201	10.1	193	12.2	190	9.6	
**Diabetes**											
Yes	848	21.3	471	56.0	149	17.5	99	12.7	129	13.7	<0.0001
No	2810	78.7	1890	68.2	324	10.5	330	12.5	266	8.9	
**Hyperlipidemia**											
Yes	1359	35.6	788	60.0	208	14.3	185	14.1	178	11.5	0.004
No	2299	64.4	1573	68.7	265	10.7	244	11.6	217	9.0	
**Asthma**											
Yes	706	17.5	363	52.5	105	14.7	103	15.3	135	17.6	<0.0001
No	2952	82.5	1998	68.4	368	11.4	326	11.9	260	8.3	
**COPD**											
Yes	432	10.9	214	49.7	61	15.1	67	16.1	90	19.1	<0.0001
No	3226	89.1	2147	67.5	412	11.6	362	12.1	305	8.8	

**GERD**											
Yes	719	18.4	382	53.2	111	15.6	105	15.0	121	16.2	<0.0001
No	2939	81.6	1979	68.4	362	11.2	324	12.0	274	8.5	

* Significant differences in osteoarthritis (OA) groups were represented by chi-square tests of likelihood ratio p-value.
COPD: Chronic obstructive pulmonary disease; GERD: Gastroesophageal reflux disease; Wt: weighted; Rx: Medication; Wid./Div./Sep.: widowed, divorced, and separated

3.2. Health-Related Quality of Life and Osteoarthritis Groups

A significant difference in the PCS and MCS scores was found between OA groups using ANOVA (Table 2). For example, OA adults with both depression and anxiety had a significantly lower mean PCS score (Mean = 36.84, SE = 0.78) and MCS score (Mean = 37.47, SE = 0.74) compared to other groups (Mean MSC score was 51.20 for OA only, 42.73 for OA and depression, and 45.71 for OA and anxiety). ANOVA test, which had shown a significant difference in all groups as P <0.0001, was followed by Tukey's Post Hoc test for multiple comparisons, which found that regarding the PCS score, adults with OA only have a significantly higher mean PCS score as compared to the other groups (OA and anxiety, OA and depression, and OA and anxiety and depression). Also, adults with OA and anxiety/depression have a significantly lower mean PCS score as compared to all groups. Regarding the MCS score, adults with OA only have a significantly higher mean MCS score as compared to the other groups. Also, adults with OA and anxiety/depression have a significantly lower mean MCS score as compared to all other groups.

Table 2. Health-related Quality of Life Weighted Means Scores by Osteoarthritis (OA) Groups

	**Total Sample**		**OA Only**		**OA & Depression**		**OA & Anxiety**		**OA & Depression & Anxiety**		
	**Mean**	**SD**	**Mean**	**SE**	**Mean**	**SE**	**Mean**	**SE**	**Mean**	**SE**	**p-value**
**HRQoL**											
**PCS**	39.91	12.43	43.12	0.32	37.39	0.79	40.91	0.78	36.84	0.78	<0.0001
**MCS**	47.21	11.00	51.20	0.22	42.73	0.61	45.71	0.61	37.47	0.74	<0.0001

Mean differences between osteoarthritis groups were analyzed using an ANOVA test.
HRQoL: Health-related Quality of Life; SE: Standard Error; SD: Standard Deviation; Sig: Significance.

> **Commented [P2]:** In summary, patients with only OA without any mental health disorder have the highest level of PCS and MCS scores (quality of life).

3.3. Health-Related Quality of Life among Osteoarthritis Groups from Linear Regression Analysis

Table 3 shows the adjusted relationship between OA groups and HRQoL. After controlling for all independent variables, OA adults with depression (MCS: β = -6.124), anxiety (MCS: β = -3.424), and both depression and anxiety (MCS: β = -10.317) had a significantly lower HRQoL mental health domain compared to those with OA only (p-value<0.0001).

OA patients, who were employed, lived in Northeast and Mid-west regions, perceived their physical health to be excellent, and exercised regularly tended to have a better quality of life. For example, OA adults who were employed have a significantly higher HRQoL in both physical health domain (PCS: β = 4.588, p-value<0.0001) and mental health domain (MCS: β = 2.858, p-value<0.0001) compared to those who were not employed.

Several factors were found to negatively impact health-related quality of life (HRQoL), including younger age, education levels, lower income, and hypertension. For instance, older adults with lower income, high school education, and hypertension had significantly lower physical health domain compared to those with high income, less than high school education, and those with hypertension (MCS: β = -1.840, p-value <0.0001). Additionally, younger older adults (aged 22-49) reported a significantly lower mental health scores (MCS: β = -3.119, p-value <0.0001) compared to those aged 50-64.

Commented [P3]: Not seems to be correct: from this table, younder adults (22-39) significantly have higher PCS compred to older adults; “not lower”. Older (40-49yrs: mid-age) not even significant!

Commented [P4]: I thought you were supposed to narrate PCS! You will need to be clear what you were describing.

Table 3. Adjusted Multivariable Linear Regressions on HRQoL among Adults with Osteoarthritis

	Health-Related Quality of Life					
	Physical Health Summary (PCS)			Mental Health Summary (MCS)		
	Regression Coefficients	SE	Sig.	Regression Coefficients	SE	Sig.
**Osteoarthritis (OA) Group**						
OA & depression	-1.042	0.716		-6.124	0.810	***
OA & anxiety	1.316	0.736		-3.424	0.617	***
OA & depression & anxiety	-0.759	0.850		-10.317	0.855	***
OA only (Ref.)						
**Age in years**						
22-39	2.398	1.029	*	-3.119	0.847	***
40-49	0.575	0.776		-2.002	0.682	**
50-64 (Ref.)						
**Gender**						
Men	0.198	0.531		-0.182	0.449	
women (Ref.)						
**Race/ethnicity**						
African American	1.133	0.728		0.832	0.631	
Latino	1.245	0.839		-1.319	0.807	
Others	-0.496	1.091		-1.404	1.108	
White (Ref.)						
**Marital status**						
Married	-0.519	0.620		0.000	0.561	
Widow/Separated/Divorce	0.471	0.693		0.591	0.642	
Never married (Ref.)						
**Education level**						
> High School	-2.203	1.385		1.469	1.433	
High School	-2.906	1.461	*	2.815	1.527	
< High School (Ref.)						
**Region**						
Northeast	1.448	0.608	*	0.029	0.665	
Mid-west	3.023	0.749	***	-0.321	0.933	
South	1.058	0.604		-0.053	0.682	
West (Ref.)						
**Employment**						
Employed	4.588	0.673	***	2.858	0.491	***
Not employed (Ref.)						
**Poverty status**						
Poor	-2.115	0.857	*	-1.205	0.853	
Near Poor	-0.684	0.842		-1.058	0.800	
Middle Income	-0.981	0.647		0.400	0.577	
High Income (Ref.)						
**Health Insurance**						

Commented [P5]: Check "Heart ???" I added the question mark to draw your attention to the hanging word…… "Heart" disease? Please, clarify.

Private	3.034	1.590		0.822	1.253	
Public	-1.696	1.702		0.711	1.233	
Uninsured (Ref.)						
**Rx Insurance**						
Rx insurance	0.863	0.833		-0.957	0.766	
No Rx insurance (Ref.)						
**General health**						
Excellent/very good	11.075	0.710	***	6.215	0.626	***
Good	6.175	0.613	***	4.004	0.616	***
Fair/poor (Ref.)						
**Physical activity**						
3/week	3.382	0.469	***	0.918	0.439	*
No exercise (Ref.)						
**Heart???**						
Yes	-0.486	0.776		-0.729	0.784	
**Hypertension**						
Yes	-1.840	0.494	***	-0.089	0.519	
**Diabetes**						
Yes	-0.928	0.549		-0.079	0.572	
**Asthma**						
Yes	-1.338	0.705		-1.175	0.703	
**COPD**						
Yes	-0.853	0.791		-0.443	1.066	
**GERD**						
Yes	-0.517	0.645		-1.162	0.641	

Asterisks denote statistical significance from multivariable linear regression on health-related quality of life among adults with osteoarthritis, ***$P < 0.001$; **$0.001 \leq P < 0.01$; *$0.01 \leq P < .05$
>: Greater than; <: Less than
COPD: Chronic obstructive pulmonary disease; GERD: Gastroesophageal reflux disease; Rx: Medication; Ref: reference group; SE: Standard Error; Sig: Significance.

4. Discussion

The findings of this study support that comorbid depression and anxiety are linked to poor HRQoL in adults with OA. Our study filled in some gaps and uncertainties about the relationship between comorbid depression and anxiety and HRQoL domains by using a nationally representative sample of US adults with OA. In our study, we compared four groups of adults with OA in terms of their quality of life: 1) those with OA only, 2) those with OA and depression, 3) those with OA and anxiety, and 4) those with OA and both depression and anxiety. By comparing these groups, we observed that those with anxiety and depression had

worse HRQoL than adults with any of these comorbidities only, or without any of them. Adults with OA and both depression and anxiety have a lower PCS score and MCS score compared to those with OA only (PCS score of 36.84 vs. 43.12, MCS score of 37.47 vs. 51.20), presence of anxiety and depression led to a nearly 6-point reduction in PCS score and a 14-point reduction in MCS score among adults with OA. In our adjusted analysis, the β-coefficient for those with both depression and anxiety (MCS: $\beta = -10.317$) indicates that having anxiety and depression comorbidity, as opposed to no mental comorbidity in adults with OA is associated with a 10-unit reduction in HRQoL score in the MCS domain. These findings indicate the incremental effect of having anxiety and depression on health status and reflect a greater disease burden of these mental illnesses. Our analysis's findings concur with those of other previously published research. For example, a study using data from the Korea National Health and Nutrition Examination Survey revealed lower HRQoL in OA adults with psychiatric disease [17]. As nearly one in ten adults with OA in our study have depression or anxiety, these findings suggest the need for OA point-of-care anxiety and depression screening [34], since they are consistently underdiagnosed and neglected. Many general practitioners and primary care providers often overlook these comorbidities because they either only address the physical aspects of OA or neglect to evaluate their patients' psychological well-being entirely [35]. Understanding these comorbidities is crucial because they can alter OA management and progression. The National Institute for Health and Care Excellence (NICE) has provided guidelines for practitioners to guide them in the holistic evaluation of patients with OA [36]. Therefore, policies and initiatives must promote and support coordinated care that covers OA and mental health management to improve their quality of life, reduce the disease burden, and fill the mental health care gap [37].

Additionally, the results of this study demonstrated that other risk factors including income, education levels, and coexisting hypertension, have an unneglectable impact on HRQoL among adults with OA. In fact, in our descriptive analysis we found that those with high school education level, low income, and those with chronic illnesses have a higher prevalence of depression and anxiety. Previous studies that involved adults in general and adults with OA reported that these are important determinants of HRQoL [6, 18, 27-33]. The negative association between having high school education and physical health (PCS domain of HRQoL), compared to less than a high school education, may be explained by the higher prevalence of depression and anxiety comorbidity from bivariate analysis in this group, as mental health conditions can exacerbate physical health issues through poor health behaviors. A recently published data from the Epidemiology of Chronic Diseases cohort study, a 10-year follow-up for 983 hip and knee OA patients reported that multimorbidity was associated with a higher risk of poor physical function and HRQoL trajectories [38]. Besides, in earlier studies, chronic illnesses are linked to mental health and are associated with a reduced HRQoL in their sufferers because of their chronic nature and management [39-41]. Given that enhancing HRQoL is seen as a noteworthy outcome and a predictor of disease management, it emphasizes the need for comprehensive care that addresses OA management and physical and mental comorbidities. Recognizing income, education, and chronic illnesses as essential factors in adults with OA can guide healthcare providers in identifying of OA individuals who may be at higher risk of experiencing poor HRQoL and tailoring interventions accordingly. For example, targeting those with low education and income with additional preventative strategies can help in mitigate the negative impact of such risk factors and prevent or minimize impairment in the quality of life of individuals with OA.

Commented [P6]: Close the paragraph

Furthermore, this study found that OA adults who were employed, lived in Northeast and Mid-west regions, perceived their physical health to be excellent, exercise regularly had higher HRQoL. Employment was particularly associated with improved physical and mental well-being, likely due to financial stability, social engagement, and a sense of purpose. Additionally, individuals with higher incomes generally experienced better health outcomes, emphasizing the role of economic security in maintaining a good quality of life. Perceived health played a crucial role in overall well-being, as individuals who rated their physical health as excellent experienced significantly better quality of life. This suggests that self-assessment of health status is a strong predictor of both physical and mental well-being. Additionally, regional differences in HRQoL were observed, with those residing in the Northeast and Midwest regions reporting better health outcomes. This could be attributed to variations in healthcare access, socioeconomic conditions, environmental factors, or community support systems available in these areas.

Physical activity also emerged as a key contributor to improved HRQoL. Prior research investigated the effects of physical activity on OA patients' physical function and HRQoL using a statewide population-based cohort reported that regular physical activity was related to enhanced physical function and HRQoL among individuals with hip and/or Knee OA [13, 42]. The results of this study further highlighted the necessity of managing OA through a multidisciplinary approach that includes lifestyle, psychological, and pharmacological interventions customized to each patient's needs to lessen the negative effects of coexisting mental conditions and, therefore, improve the overall quality of life.

4.1. Study Strengths and Limitations

This study used a large sample size and a nationally representative sample of US adults with OA, which allowed us to obtain a clinically insightful estimate of the relationship between depression

and anxiety and HRQoL in adults with OA. However, this study has some limitations. First, the cross-sectional design of our study limits its ability to determine the causal relationship between depression, anxiety, and HRQoL. Second, specific critical confounders were not available on MEPS data and have not been adjusted for in the analysis, including osteoarthritis-related variables (i.e., treatment, the severity of their illness, disease subtypes, OA-related pain, pain-severity, and pain location (hip vs. knee joint)) which can interfere with a person's ability to function normally and have a negative relationship with HRQoL [17, 38, 43, 44]. These essential confounders were overlooked, which all have a significant role in influencing HRQoL in adults with OA and are crucial to comprehending the intricate relationship between mental health and OA patients' HRQoL. Third, missing these critical OA-related variables in the analysis may have introduced a potential omitted variable bias and weakened the study's findings and conclusion; thus, the results of this study may have overestimated the direct impact of anxiety and depression on HRQoL in persons with osteoarthritis, therefore, adding these crucial factors should be the top priority for future research. However, in this study's analysis compared to previously published research, an adjustment for several essential confounders that can potentially affect the HRQoL, such as social determinants, chronic illnesses, physical activity, health insurance coverage, and perceived health, were included to produce greater confidence in detecting the actual effect of mental health on HRQoL in adults with OA [27-33]. Finally, the fact that data on HRQoL and OA symptoms are mostly self-reported introduces a potential source for biases such as underreporting or misreporting bias.

4.2. Clinical Practice, Policy and Public Health Implications

The clinical practice implication of the results of our research should reassure us that physicians, rheumatologists, and mental health professionals should incorporate routine screening for

anxiety and depression in adults with OA, especially when patients or their families report symptoms [45]. This can help minimize suffering, improve symptom management, and enhance HRQoL. Clinicians should adhere to the NICE guidelines on identifying and treating depression, utilizing a combination of pharmacotherapy, cognitive-behavioral therapy (CBT), physical therapies, and structured activities to optimize treatment outcomes [46]. Some research suggested that the use of CBT, education programs, pharmacotherapy, and physical exercise are linked to the short-term reduction of depressed symptoms in older people with OA [35, 47]. Besides, healthcare providers should adopt an integrated pain management approach, combining pharmacologic treatments (e.g., simple analgesics, NSAIDs, COX-2 inhibitors) with non-pharmacologic interventions (e.g., movement meditation, acupuncture, and psychological therapy) to reduce pain-related anxiety and depression in OA patients [48-50].

The policy implications of this study suggest that policymakers should develop and enforce guidelines that promote the integration of mental health screening into routine OA care at primary and specialty healthcare settings [34]. Governments and health insurance providers must enhance access to affordable mental health services by reducing financial barriers for uninsured and underinsured populations. This could include expanding publicly funded mental health programs and increasing coverage for psychological services in health insurance plans. This is especially important due to the high cost of mental health care, as people with mental illnesses have historically faced major barriers to seeking mental health care for the uninsured and even those who are insured [51].

The public health implications encourage lifestyle changes like physical activity. Physical activity equivalent to 150 minutes/week of moderate-intensity exercise or at least 45 minutes/week of moderate-intensity can have a detrimental positive impact on HRQoL [42, 52,

Commented [P7]: Likely incorrect… consider: “least **150 minutes per week** of moderate-intensity aerobic activity or 75 minutes per week of vigorous aerobic activity, or a combination of both.” Source: https://www.heart.org/en/healthy-living/fitness/fitness-basics/aha-recs-for-physical-activity-in-adults

53], decreased OA pain [53], improve physical function of OA patients [52, 53]. Public health agencies should leverage exercise-based interventions as a cost-effective alternative to antidepressant treatment, given its comparable benefits in reducing depression and anxiety in OA patients [54, 55].

5. Conclusions

The relationships between anxiety, depression, and a low HRQoL in patients with OA was found in this study. This study also revealed the negative effect of low education and low income on HRQoL while highlighting the advantages of employment and regular exercise. It is crucial for future research to thoroughly evaluate patient-centered clinical approaches ~~and,~~ care and management policies. This understanding of risk factors that hinder patients with OA, which increase anxiety and depression risk and therefore compromise the HRQoL in individuals with OA, is worthy.

Formatted: Font color: Red, Strikethrough

Formatted: Font color: Red

List of Abbreviations

HRQoL Health-Related Quality of Life, MCS mental health component score, PCS physical health component score.

Declarations

Funding

Authors' Contributions

MA: design of the study, searching the literature, manuscript writing, and analyzing the findings; TA: searching the literature, and manuscript writing. Both authors read and approved the final manuscript.

Institutional Review Board Statement

No ethical review or approval was needed as we have utilized a publicly accessible, the Medical Expenditure Panel Survey (MEPS) database.

Informed Consent Statement

Medical Expenditure Panel Survey (MEPS) database is publicly accessible secondary data, therefore, patient consent was waived for this research.

Data Availability Statement

The dataset used in this study is available from the MEPS database at this URL: https://meps.ahrq.gov/data_stats.

Conflicts of Interest

The author affirms that there are no conflicts of interest related to the publication of this paper

References

1. Leifer VP, Katz JN, Losina E. The burden of OA-health services and economics. Osteoarthritis and Cartilage. 2022;30(1):10-6.

2. Steinmetz JD, Culbreth GT, Haile LM, Rafferty Q, Lo J, Fukutaki KG, et al. Global, regional, and national burden of osteoarthritis, 1990–2020 and projections to 2050: a systematic analysis for the Global Burden of Disease Study 2021. The Lancet Rheumatology. 2023;5(9):e508-e22.

3. Allen KD, Thoma LM, Golightly YM. Epidemiology of osteoarthritis. Osteoarthritis and Cartilage. 2022;30(2):184-95.

4. Scheuing WJ, Reginato AM, Deeb M, Acer Kasman S. The burden of osteoarthritis: Is it a rising problem? Best Practice & Research Clinical Rheumatology. 2023;37(2):101836.

5. Rothrauff B, Tang Q, Wang J, He J. Osteoarthritis is positively associated with self-reported sleep trouble in older adults. Aging Clinical and Experimental Research. 2022;34(11):2835-43.

6. Chang W-Y, Choi S, Yoo SJ, Lee J, Lim C. Factors Associated with Osteoarthritis and Their Influence on Health-Related Quality of Life in Older Adults with Osteoarthritis: A Study Based on the 2020 Korea National Health and Nutrition Examination Survey. International Journal of Environmental Research and Public Health. 2023;20(12):6073.

7. Zhao X, Shah D, Gandhi K, Wei W, Dwibedi N, Webster L, et al. Clinical, humanistic, and economic burden of osteoarthritis among noninstitutionalized adults in the United States. Osteoarthritis and Cartilage. 2019;27(11):1618-26.

8. Savvari P, Skiadas I, Papadakis SA, Psychogios V, Argyropoulou OD, Pastroudis AP, et al. The impact of moderate to severe osteoarthritis on the physical performance and quality of

life: a cross-sectional study in Greek patients (PONOS study). BMC Musculoskeletal Disorders. 2023;24(1):651.

9. Jin X, Liang W, Zhang L, Cao S, Yang L, Xie F. Economic and humanistic burden of osteoarthritis: an updated systematic review of large sample studies. Pharmacoeconomics. 2023;41(11):1453-67.

10. Yan H, Guo J, Zhou W, Dong C, Liu J. Health-related quality of life in osteoarthritis patients: a systematic review and meta-analysis. Psychology, Health & Medicine. 2022;27(8):1859-74.

11. Wang ST, Ni GX. Depression in Osteoarthritis: Current Understanding. Neuropsychiatr Dis Treat. 2022;18:375-89.

12. Sambamoorthi U, Shah D, Zhao X. Healthcare burden of depression in adults with arthritis. Expert Rev Pharmacoecon Outcomes Res. 2017;17(1):53-65.

13. Fonseca-Rodrigues D, Rodrigues A, Martins T, Pinto J, Amorim D, Almeida A, et al. Correlation between pain severity and levels of anxiety and depression in osteoarthritis patients: a systematic review and meta-analysis. Rheumatology. 2021;61(1):53-75.

14. Rosemann T, Gensichen J, Sauer N, Laux G, Szecsenyi J. The impact of concomitant depression on quality of life and health service utilisation in patients with osteoarthritis. Rheumatology International. 2007;27(9):859-63.

15. Tsuji T, Nakata K, Vietri J, Jaffe DH. The added burden of depression in patients with osteoarthritis in Japan. ClinicoEconomics and Outcomes Research. 2019;11:411-21.

16. Agarwal P, Sambamoorthi U. Healthcare Expenditures Associated with Depression Among Individuals with Osteoarthritis: Post-Regression Linear Decomposition Approach. Journal of General Internal Medicine. 2015;30(12):1803-11.

17. Lee Y, Lee S-H, Lim SM, Baek SH, Ha I-H. Mental health and quality of life of patients with osteoarthritis pain: The sixth Korea National Health and Nutrition Examination Survey (2013–2015). PLOS ONE. 2020;15(11):e0242077.

18. Kim DJ. A Study on the Physical Activities, Mental Health, and Health-Related Quality of Life of Osteoarthritis Patients. Osong Public Health Res Perspect. 2019;10(6):368-75.

19. Karimi M, Brazier J. Health, Health-Related Quality of Life, and Quality of Life: What is the Difference? PharmacoEconomics. 2016;34(7):645-9.

20. Pinto Barbosa S, Marques L, Sugawara A, Toledo F, Imamura M, Battistella L, et al. Predictors of the Health-Related Quality of Life (HRQOL) in SF-36 in Knee Osteoarthritis Patients: A Multimodal Model With Moderators and Mediators. Cureus. 2022;14(7):e27339.

21. Tavares DRB, Moça Trevisani VF, Frazao Okazaki JE, Valéria de Andrade Santana M, Pereira Nunes Pinto AC, Tutiya KK, et al. Risk factors of pain, physical function, and health-related quality of life in elderly people with knee osteoarthritis: A cross-sectional study. Heliyon. 2020;6(12):e05723.

22. Agency for Healthcare Research and Quality. Medical Expenditure Panel Survey HC-231: 2021 Medical Conditions File. [cited 2024]; Available from: https://meps.ahrq.gov/data_stats/download_data/pufs/h231/h231doc.pdf.

23. Maslak JP, Jenkins TJ, Weiner JA, Kannan AS, Patoli DM, McCarthy MH, et al. Burden of Sciatica on US Medicare Recipients. JAAOS - Journal of the American Academy of Orthopaedic Surgeons. 2020;28(10):e433-e9.

24. Selim AJ, Rothendler JA, Qian SX, Bailey HM, Kazis LE. The History and Applications of the Veterans RAND 12-Item Health Survey (VR-12). The Journal of Ambulatory Care Management. 2022;45(3):161-70.

25. Kazis LE, Rogers WH, Rothendler J, Qian S, Selim A, Edelen MO, et al. Outcome Performance Measure Development for Persons with Multiple Chronic Conditions. Santa Monica, CA: RAND Corporation; 2017.

26. Cheak-Zamora NC, Wyrwich KW, McBride TD. Reliability and validity of the SF-12v2 in the medical expenditure panel survey. Quality of Life Research. 2009;18(6):727-35.

27. Heyworth ITM, Hazell ML, Linehan MF, Frank TL. How do common chronic conditions affect health-related quality of life? British Journal of General Practice. 2009;59(568):e353-8.

28. Keles H, Ekici A, Ekici M, Bulcun E, Altinkaya V. Effect of chronic diseases and associated psychological distress on health-related quality of life. Internal Medicine Journal. 2007;37(1):6-11.

29. Kivits J, Erpelding ML, Guillemin F. Social determinants of health-related quality of life. Rev Epidemiol Sante Publique. 2013;61 Suppl 3:S189-94.

30. Saffari M, Meybodi MKE, Ghanizadeh G, Koenig HG. Determinants of health status among patients with knee or hip osteoarthritis: the role of demographic, clinical and health related quality of life variables. Journal of Musculoskeletal Research. 2014;17(01):1450004.

31. Yuniarwati W, Subijanto AA, Prasetyo H. Determinants of Quality of Life among Patient with Knee Osteoarthritis. Journal of Epidemiology and Public Health. 2019;4(4):320-7.

32. Luong M-LN, Cleveland RJ, Nyrop KA, Callahan LF. Social Determinants and Osteoarthritis Outcomes. Aging Health. 2012;8(4):413-37.

33. Bharmal M, Thomas J, 3rd. Health insurance coverage and health-related quality of life: analysis of 2000 Medical Expenditure Panel Survey data. J Health Care Poor Underserved. 2005;16(4):643-54.

34. Mallen CD, Nicholl BI, Lewis M, Bartlam B, Green D, Jowett S, et al. The effects of implementing a point-of-care electronic template to prompt routine anxiety and depression screening in patients consulting for osteoarthritis (the Primary Care Osteoarthritis Trial): A cluster randomised trial in primary care. PLoS Med. 2017;14(4):e1002273.

35. Sharma A, Kudesia P, Shi Q, Gandhi R. Anxiety and depression in patients with osteoarthritis: impact and management challenges. Open Access Rheumatology: Research and Reviews. 2016;8:103-13.

36. National Institute for Health and Care Excellence. Osteoarthritis in over 16s: diagnosis and management. 2022; Available from: https://www.nice.org.uk/guidance/ng226/resources/osteoarthritis-in-over-16s-diagnosis-and-management-pdf-66143839026373.

37. Gleicher Y, Croxford R, Hochman J, Hawker G. A prospective study of mental health care for comorbid depressed mood in older adults with painful osteoarthritis. BMC Psychiatry. 2011;11:147.

38. Costa D, Lopes DG, Cruz EB, Henriques AR, Branco J, Canhão H, et al. Trajectories of physical function and quality of life in people with osteoarthritis: results from a 10-year population-based cohort. BMC Public Health. 2023;23(1):1407.

39. Park Y, Park K. Health-related quality of life and depressive symptoms of patients with chronic diseases and the general population before and during the COVID-19 pandemic in Korea. Front Psychol. 2023;14:1117369.

40. Maynié-François C, Burtey S. Comparing health-related quality of life in chronic diseases: the importance of analyzing references. Nature Reviews Disease Primers. 2019;5(1): e27.

41. Megari K. Quality of Life in Chronic Disease Patients. Health Psychol Res. 2013;1(3):24.

42. Anokye NK, Trueman P, Green C, Pavey TG, Taylor RS. Physical activity and health related quality of life. BMC Public Health. 2012;12:624.

43. Chang J, Yuan Y, Fu M, Wang D. Health-related quality of life among patients with knee osteoarthritis in Guangzhou, China: a multicenter cross-sectional study. Health and Quality of Life Outcomes. 2023;21(1):50.

44. Montero A, Mulero J-F, Tornero C, Guitart J, Serrano M. Pain, disability and health-related quality of life in osteoarthritis—joint matters: an observational, multi-specialty trans-national follow-up study. Clinical Rheumatology. 2016;35(9):2293-305.

45. Frankish H, Boyce N, Horton R. Mental health for all: a global goal. The Lancet. 2018;392(10157):1493-4.

46. National Institute for Health and Care Excellence. Depression in adults: treatment and management. 2022; Available from: https://www.nice.org.uk/guidance/ng222.

47. Yohannes AM, Caton S. Management of depression in older people with osteoarthritis: A systematic review. Aging & Mental Health. 2010;14(6):637-51.

48. Burley CV, Casey A-N, Jones MD, Wright KE, Parmenter BJ. Nonpharmacological approaches for pain and symptoms of depression in people with osteoarthritis: systematic review and meta-analyses. Scientific Reports. 2023;13(1):15449.

49. Manyanga T, Froese M, Zarychanski R, Abou-Setta A, Friesen C, Tennenhouse M, et al. Pain management with acupuncture in osteoarthritis: a systematic review and meta-analysis. BMC Complementary and Alternative Medicine. 2014;14:312.

50. Walker-Bone K, Javaid K, Arden N, Cooper C. Medical management of osteoarthritis. BMJ. 2000;321(7266):936-40.

51. Rowan K, McAlpine DD, Blewett LA. Access and cost barriers to mental health care, by insurance status, 1999-2010. Health Aff. 2013;32(10):1723-30.

52. Lopes DG, Costa D, Cruz EB, Mendonça N, Henriques AR, Branco J, et al. Association of physical activity with physical function and quality of life in people with hip and knee osteoarthritis: longitudinal analysis of a population-based cohort. Arthritis Research & Therapy. 2023;25(1):14.

53. Kraus VB, Sprow K, Powell KE, Buchner D, Bloodgood B, Piercy K, et al. Effects of Physical Activity in Knee and Hip Osteoarthritis: A Systematic Umbrella Review. Med Sci Sports Exerc. 2019;51(6):1324-39.

54. Dinas PC, Koutedakis Y, Flouris AD. Effects of exercise and physical activity on depression. Irish Journal of Medical Science. 2011;180(2):319-25.

55. Kandola A, Ashdown-Franks G, Hendrikse J, Sabiston CM, Stubbs B. Physical activity and depression: Towards understanding the antidepressant mechanisms of physical activity. Neuroscience & Biobehavioral Reviews. 2019;107:525-39.

Revised Manuscript with Track Changes

Mental Health and Health-Related Quality of Life among Adults with Osteoarthritis: A National Population-Based Study

Running Title: Depression and Anxiety and Quality of Life among Osteoarthritis

Monira Alwhaibi[1,2*¶] Tariq M. Alhawassi[1,2¶]

[1]Department of Clinical Pharmacy, College of Pharmacy, King Saud University, Riyadh, Saudi Arabia
[2] Medication Safety Research Chair, College of Pharmacy, King Saud University, Riyadh, Saudi Arabia

¶These authors contributed equally to this work.

*Corresponding author: Monira Alwhaibi, MS, PhD, Professor, Department of Clinical Pharmacy, King Saud University, College of Pharmacy, Riyadh, 11149, Saudi Arabia, E-mail: malwhaibi@ksu.edu.sa

ORCID ID Corresponding Author: 0000-0003-3707-0582

Abstract

Background: Adults with osteoarthritis are vulnerable to mental health disorders, which may have a significant impact on their Health-Related Quality of Life (HRQoL). Thus, the purpose of this study was to look at the relationship between anxiety and depression and HRQoL in adults with osteoarthritis.

Methods: Adult patients diagnosed with osteoarthritis who were 18 years of age or older were identified using data from the Medical Expenditure Panel Survey for the years 2018 through 2021. HRQoL was computed using the veteran's RAND 12-item health survey which has two domains the Physical and Mental Component Summary (PCS & MCS) ratings. The link between anxiety and depression and HRQoL in individuals with osteoarthritis was examined using multivariable linear regression after the adjustment for a variety of covariates. **Results:** Of the 3,658 individuals with osteoarthritis that were identified, 12.0% experienced depression, 12.5% had anxiety, and 9.9% had both illnesses. The PCS and MCS mean scores of the HRQoL were lowest for persons with comorbid depression and anxiety compared to adults with osteoarthritis only. Moreover, from the adjusted regression analysis, adults with osteoarthritis who had depression (MCS: adjusted $\beta = -5.408$, $p<0.001$), anxiety (MCS: adjusted $\beta = -3.485$, $p<0.001$), or both depression and anxiety (MCS: adjusted $\beta = -10.348$, p-value<0.0001) had significantly lower HRQoL MCS scores compared to those with osteoarthritis only. Those who were physically active and employed had notably better scores on the PCS and MCS than those who were not.

Conclusions: This nationally representative sample has shed light on the relationships between anxiety, depression, and a low HRQoL in adults with osteoarthritis. The study also revealed the impact of socioeconomic factors such as education level and income on HRQoL, and the benefits of employment and regular exercise. Importantly, the findings underscore the need for lifestyle modifications to reduce anxiety and depression in individuals with osteoarthritis. They also highlight the practical implications for healthcare planning and resource allocation, providing valuable insights for policymakers and healthcare professionals.

Keywords. Anxiety; Osteoarthritis; Depression; Quality of Life; Rand 12.

1. Introduction

Osteoarthritis (OA) is one of the most prevalent disabling illnesses globally and has been shown to have serious adverse effects on health [1]. It was estimated that OA affected around six hundred million individuals worldwide in 2020, representing 7.6% of the world's population [2]. Moreover, OA cases are expected to rise by nearly 50% for the hand, 75% for the knee and the hip, and up to 95% increase in OA prevalence for other types by 2050 as compared to 2020 [2]. Nowadays, OA is the 14th leading cause of age-standardized years lived with disability (YLDs) globally and has a substantial burden on individuals characterized by pain, limitations of activity, and diminished quality of life [2]. In addition to the direct health-related burden, OA has a significant financial burden and detrimental effects on a person's psychological health and well-being, work participation, sleep, mobility, and diminished health-related quality of life (HRQoL) [1, 3-10]. Therefore, individuals with OA have three times more potential than individuals in the general population to experience anxiety and depression, which could be attributed to pain severity, poor function, number of OA sites, immunological inflammation, and structural alterations in the brain [11-13].

Anxiety and depression can increase the risk of adverse clinical outcomes, such as OA illness burden and morbidity, and also result in increased healthcare costs and health service utilization, with higher healthcare provider visits, emergency room visits, and hospitalizations [12, 14-16]. Besides, depression was associated with lower HRQoL for individuals with OA [17, 18]. HRQoL is a crucial outcome measure for evaluating the overall effects of long-term illnesses like OA, including a person's social, emotional, and physical well-being [19].

With the aforementioned risk of OA on patients' psychological well-being, maintaining a high quality of life in terms of health despite the difficulties posed by OA is a primary objective

for adult OA sufferers and their healthcare providers. Therefore, understanding the causes underlying a lower HRQoL becomes essential, especially when considering mental health. Yet, the relationship between depression and anxiety and the HRQoL of people with OA is not fully understood, given that the number of studies that evaluated this relationship is limited either in terms of generalizability (most of the published research was conducted in Japan and Korea) or limited in terms of methods used in identifying associations [6, 12, 15, 17-21]. Consequently, to optimize OA patient health, healthcare providers must have a thorough understanding of the mutual interaction that exists between depression, anxiety, and the general impact on HRQoL among such vulnerable populations. Therefore, this led us to conduct this research, which aimed at evaluating the association between depression, anxiety, and the HRQoL among adults with OA using a sample from a representative United States (US) database and adding more insightful knowledge to the currently available research in this significant field.

2. Methods

2.1. Study Design and Data

This study used Data from the Medical Expenditure Panel Survey (MEPS) from 2018 to 2021 to conduct a cross-sectional study. MEPS is a nationwide survey that gathers information on sociodemographics, health insurance, medical issues, medication use, and medical care from United States (US) citizens.

2.2. Study Population

According to the study's inclusion criteria, an adult must be between 18 and 64 years old, have a diagnosis of OA, alive within the study period, and have no missing data on HRQoL. The International Classification of Diseases, tenth Revision, Clinical Modification (ICD-10-CM)

clinical diagnosis codes were used to identify OA from MEPS data (M16: osteoarthritis of the hip, M17: osteoarthritis of the knee, M19: other and unspecified osteoarthritis) [22].

2.3. Measures

2.3.1. Outcome: Health-Related Quality of Life (HRQoL)

To evaluate the HRQoL, adults who participated in the MEPS filled out a questionnaire using a reliable and valid instrument, the Veterans RAND 12 Item Health Survey (VR-12©) [23-25]. VR-12 is a 12-item patient-reported outcome measure that evaluates eight domains of health: general health, physical functioning, role physical, bodily pain, vitality, role emotional, mental health, and social functioning [23]. The VR-12 has two parts: 1/ Physical Component Summary (PCS), which included responses from the general health, physical functioning, role physical, and bodily pain domains, and 2/ Mental Component Summary (MCS), which included responses from role emotional, vitality, mental health, and social functioning domains [26]. Scores range from 0 to 100, with higher PCS and MCS scores as better physical and mental HRQoL indicators.

2.4.2. Independent Variables

The primary independent variable of interest in this study was the different OA groups, each of which was mutually exclusive (OA only, OA and anxiety, OA and depression, and OA and both mental illnesses). Other independent variables were selected based on their documented influence on HRQoL in adults, both in general and those with OA, as reported in the literature including the social determinants (e.g., age, gender, education, occupational status, household income), chronic illnesses, physical activity, health insurance coverage, and perceived health [27-33].

2.5. Statistical Analyses

Descriptive statistics were employed to show the study sample's baseline characteristics, including mean, standard deviation, frequencies, and percentages. The differences in individual characteristics between the OA groups were evaluated using chi-square tests. The mean variances in HRQoL by OA groups were determined using ANOVA followed by Tukey's post hoc test for multiple comparisons to identify which specific groups differed. To assess the link between OA groups and HRQoL, we used multivariable linear regression and adjusted for all independent covariates. Variables were included in the model based on their theoretical relevance, clinical importance, and prior literature supporting their association with health-related quality of life (HRQoL) in OA patients [27-33]. Additionally, a bivariate analysis was performed and only variables with a likelihood ratio p-value of <0.05 were considered for the multivariable regression model. All the underlying assumptions of the linear regression model have been evaluated to ensure that they are satisfied by the regression model and are met by the variables. Those assumptions include correct model specification, normality, homoscedasticity, no autocorrelation, and no multicollinearity. After checking the underlying assumptions, we performed the linear regression model. All estimations in the statistical analysis considered the complicated survey design of the MEPS by integrating variance adjustment weights (strata and primary sample unit) from the MEPS with person-level weights. The Statistical Analysis System, SAS 9.4 (SAS Institute Inc., Cary, NC, USA), was used to analyze the data.

3. Results

3.1. Characteristics of the Study Sample

Table 1 lists the characteristics of the study sample, 3,658 adults with OA; the majority of the study sample was women (62.3%), aged 50 to 64 (72.0%), White (73.3%), and had higher than

high school (87.8%). About 12.0% of adults with OA also experienced depression, 12.5% had anxiety, and 9.9% had both illnesses.

Women with OA showed significantly higher rates of depression (13.6% vs. 9.3%), anxiety (14.7% vs. 9.0%), and comorbid anxiety and depression (12.0% vs. 6.5%, p-value = <0.0001) when compared to men. Furthermore, unemployed OA patients had significantly higher rates of depression (16.2% vs. 9.3%) and comorbid anxiety and depression (13.7% vs. 7.5%, p-value <0.0001) than employed OA patients. OAs adults with diabetes, asthma, COPD, and GERD had significantly higher rates of comorbid depression and anxiety than those without these comorbidities (p-value <0.0001).

Table 1. Characteristics of the Study Sample, n=3,658 Adults with Osteoarthritis (OA), Number and Row % of Sample Characteristics by OA Groups

	Total Sample		OA Only		OA & Depression		OA & Anxiety		OA & Depression & Anxiety		
	N	Wt.%	N	Wt.%	N	Wt.%	N	Wt.%	N	Wt.%	P-value*
**All**	3,658	100.0	2361	65.6	473	12.0	429	12.5	395	9.9	
**Age in years**											
22-39	327	9.7	187	54.4	31	10.8	52	16.8	57	18.0	<0.0001
40-49	648	18.2	384	62.4	87	12.7	90	13.4	87	11.5	
50-64	2683	72.0	1790	67.9	355	12.0	287	11.7	251	8.4	
**Gender**											
Women	2405	62.3	1437	59.8	336	13.6	319	14.7	313	12.0	<0.0001
Men	1253	37.7	924	75.2	137	9.3	110	9.0	82	6.5	
**Race/ethnicity**											
White	2304	73.3	1412	63.5	318	12.4	305	13.8	269	10.2	0.011
African American	658	12.5	482	73.6	66	9.5	62	8.5	48	8.4	
Latino	488	8.8	336	72.1	58	11.3	42	8.1	52	8.5	
Others	208	5.4	131	64.9	31	13.0	20	11.1	26	11.0	
**Marital status**											
Married	1749	54.8	1232	71.0	190	10.3	199	12.2	128	6.5	<0.0001
Widow/Sep/Div	1250	30.2	712	57.6	200	14.1	166	13.9	172	14.4	
Never married	659	15.0	417	62.1	83	13.7	64	11.0	95	13.2	
**Education level**											
< High School	197	3.4	133	70.8	24	12.2	22	9.8	18	7.2	0.015
High School	395	8.0	240	61.7	54	13.2	38	8.7	63	16.5	
> High School	3033	87.8	1965	65.7	392	11.9	365	13.0	311	9.4	

**Region**											
Northeast	589	16.9	385	68.3	70	9.4	57	10.1	77	12.2	0.356
Mid-west	939	26.3	572	63.4	145	13.9	113	12.2	109	10.4	
South	1444	38.8	946	64.9	169	12.5	175	13.1	154	9.5	
West	686	18.0	458	67.7	89	10.5	84	14.1	55	7.7	
**Employment**											
Employed	1942	60.8	1399	72.0	192	9.3	198	11.3	153	7.5	<0.0001
Not employed	1716	39.2	962	55.7	281	16.2	231	14.4	242	13.7	
**Poverty status**											
Poor	814	15.8	441	50.7	127	15.8	114	15.5	132	18.0	<0.0001
Near Poor	723	16.0	423	57.7	112	16.8	93	13.3	95	12.2	
Middle Income	911	24.8	606	66.4	123	12.7	101	12.4	81	8.6	
High Income	1210	43.5	891	73.5	111	8.4	121	11.2	87	6.8	
**Health Insurance**											
Private	2098	67.0	1507	71.9	227	9.8	210	11.2	154	7.1	<0.0001
Public	1415	29.4	755	51.3	227	16.6	207	15.7	226	16.4	
Uninsured	145	3.6	99	65.5	19	14.0	12	11.2	15	9.3	
**Rx Insurance**											
Rx insurance	1850	59.4	1347	72.8	185	9.3	187	11.1	131	6.8	<0.0001
No Rx insurance	1808	40.6	1014	55.1	288	15.9	242	14.5	264	14.4	
**General health**											
Excellent/very good	1002	31.3	770	77.2	100	8.5	96	10.9	36	3.4	<0.0001
Good	1302	36.8	876	66.8	154	11.7	146	11.9	126	9.6	
Fair/poor	1354	31.9	715	52.8	219	15.8	187	14.8	233	16.6	
**Physical activity**											
3/week	1496	43.1	1054	71.2	169	9.8	156	12.0	117	7.0	<0.0001
No exercise	2158	56.8	1305	61.4	302	13.6	273	12.9	278	12.1	
**Heart**											
Yes	422	10.5	235	58.6	63	14.7	62	13.8	62	12.8	0.111
No	3236	89.5	2126	66.4	410	11.7	367	12.4	333	9.5	
**Hypertension**											
Yes	1820	45.9	1107	62.6	272	14.2	236	12.9	205	10.2	0.017
No	1838	54.1	1254	68.2	201	10.1	193	12.2	190	9.6	
**Diabetes**											
Yes	848	21.3	471	56.0	149	17.5	99	12.7	129	13.7	<0.0001
No	2810	78.7	1890	68.2	324	10.5	330	12.5	266	8.9	
**Hyperlipidemia**											
Yes	1359	35.6	788	60.0	208	14.3	185	14.1	178	11.5	0.004
No	2299	64.4	1573	68.7	265	10.7	244	11.6	217	9.0	
**Asthma**											
Yes	706	17.5	363	52.5	105	14.7	103	15.3	135	17.6	<0.0001
No	2952	82.5	1998	68.4	368	11.4	326	11.9	260	8.3	
**COPD**											
Yes	432	10.9	214	49.7	61	15.1	67	16.1	90	19.1	<0.0001
No	3226	89.1	2147	67.5	412	11.6	362	12.1	305	8.8	

**GERD**											
Yes	719	18.4	382	53.2	111	15.6	105	15.0	121	16.2	<0.0001
No	2939	81.6	1979	68.4	362	11.2	324	12.0	274	8.5	

* Significant differences in osteoarthritis (OA) groups were represented by chi-square tests of likelihood ratio p-value.
COPD: Chronic obstructive pulmonary disease; GERD: Gastroesophageal reflux disease; Wt: weighted; Rx: Medication; Wid./Div./Sep.: widowed, divorced, and separated

3.2. Health-Related Quality of Life and Osteoarthritis Groups

A significant difference in the PCS and MCS scores was found between OA groups using ANOVA (Table 2). For example, OA adults with both depression and anxiety had a significantly lower mean PCS score (Mean = 36.84, SE = 0.78) and MCS score (Mean = 37.47, SE = 0.74) compared to other groups (Mean MSC score was 51.20 for OA only, 42.73 for OA and depression, and 45.71 for OA and anxiety). ANOVA test, which had shown a significant difference in all groups as P <0.0001, was followed by Tukey's Post Hoc test for multiple comparisons, which found that regarding the PCS score, adults with OA only have a significantly higher mean PCS score as compared to the other groups (OA and anxiety, OA and depression, and OA and anxiety and depression). Also, adults with OA and anxiety/depression have a significantly lower mean PCS score as compared to all groups. Regarding the MCS score, adults with OA only have a significantly higher mean MCS score as compared to the other groups. Also, adults with OA and anxiety/depression have a significantly lower mean MCS score as compared to all other groups.

Table 2. Health-related Quality of Life Weighted Means Scores by Osteoarthritis (OA) Groups

	**Total Sample**		**OA Only**		**OA & Depression**		**OA & Anxiety**		**OA & Depression & Anxiety**		
	**Mean**	**SD**	**Mean**	**SE**	**Mean**	**SE**	**Mean**	**SE**	**Mean**	**SE**	**p-value**
**HRQoL**											
**PCS**	39.91	12.43	43.12	0.32	37.39	0.79	40.91	0.78	36.84	0.78	<0.0001
**MCS**	47.21	11.00	51.20	0.22	42.73	0.61	45.71	0.61	37.47	0.74	<0.0001

Mean differences between osteoarthritis groups were analyzed using an ANOVA test.
HRQoL: Health-related Quality of Life; SE: Standard Error; SD: Standard Deviation; Sig: Significance.

3.3. Health-Related Quality of Life among Osteoarthritis Groups from Linear Regression Analysis

Table 3 shows the adjusted relationship between OA groups and HRQoL. After controlling for all independent variables, OA adults with depression (MCS: β = -6.124), anxiety (MCS: β = -3.424), and both depression and anxiety (MCS: β = -10.317) had a significantly lower HRQoL mental health domain compared to those with OA only (p-value<0.0001).

OA patients, who were employed, lived in Northeast and Mid-west regions, perceived their physical health to be excellent, and exercised regularly tended to have a better quality of life. For example, OA adults who were employed have a significantly higher HRQoL in both physical health domain (PCS: β = 4.588, p-value<0.0001) and mental health domain (MCS: β = 2.858, p-value<0.0001) compared to those who were not employed.

Several factors were found to negatively impact health-related quality of life (HRQoL), including younger age, education levels, lower income, and hypertension. For instance, older adults with lower income, high school education, and hypertension had significantly lower physical health domain compared to those with high income, less than high school education, and those with hypertension (MCS: β = -1.840, p-value <0.0001). Additionally, younger older adults (aged 22-49) reported a significantly lower mental health scores (MCS: β = -3.119, p-value <0.0001) compared to those aged 50-64.

Table 3. Adjusted Multivariable Linear Regressions on HRQoL among Adults with Osteoarthritis

	Health-Related Quality of Life					
	Physical Health Summary (PCS)			Mental Health Summary (MCS)		
	Regression Coefficients	SE	Sig.	Regression Coefficients	SE	Sig.
**Osteoarthritis (OA) Group**						
OA & depression	-1.042	0.716		-6.124	0.810	***
OA & anxiety	1.316	0.736		-3.424	0.617	***
OA & depression & anxiety	-0.759	0.850		-10.317	0.855	***
OA only (Ref.)						
**Age in years**						
22-39	2.398	1.029	*	-3.119	0.847	***
40-49	0.575	0.776		-2.002	0.682	**
50-64 (Ref.)						
**Gender**						
Men	0.198	0.531		-0.182	0.449	
women (Ref.)						
**Race/ethnicity**						
African American	1.133	0.728		0.832	0.631	
Latino	1.245	0.839		-1.319	0.807	
Others	-0.496	1.091		-1.404	1.108	
White (Ref.)						
**Marital status**						
Married	-0.519	0.620		0.000	0.561	
Widow/Separated/Divorce	0.471	0.693		0.591	0.642	
Never married (Ref.)						
**Education level**						
> High School	-2.203	1.385		1.469	1.433	
High School	-2.906	1.461	*	2.815	1.527	
< High School (Ref.)						
**Region**						
Northeast	1.448	0.608	*	0.029	0.665	
Mid-west	3.023	0.749	***	-0.321	0.933	
South	1.058	0.604		-0.053	0.682	
West (Ref.)						
**Employment**						
Employed	4.588	0.673	***	2.858	0.491	***
Not employed (Ref.)						
**Poverty status**						
Poor	-2.115	0.857	*	-1.205	0.853	
Near Poor	-0.684	0.842		-1.058	0.800	
Middle Income	-0.981	0.647		0.400	0.577	
High Income (Ref.)						
**Health Insurance**						

Private	3.034	1.590		0.822	1.253	
Public	-1.696	1.702		0.711	1.233	
Uninsured (Ref.)						
**Rx Insurance**						
Rx insurance	0.863	0.833		-0.957	0.766	
No Rx insurance (Ref.)						
**General health**						
Excellent/very good	11.075	0.710	***	6.215	0.626	***
Good	6.175	0.613	***	4.004	0.616	***
Fair/poor (Ref.)						
**Physical activity**						
3/week	3.382	0.469	***	0.918	0.439	*
No exercise (Ref.)						
**Heart**						
Yes	-0.486	0.776		-0.729	0.784	
**Hypertension**						
Yes	-1.840	0.494	***	-0.089	0.519	
**Diabetes**						
Yes	-0.928	0.549		-0.079	0.572	
**Asthma**						
Yes	-1.338	0.705		-1.175	0.703	
**COPD**						
Yes	-0.853	0.791		-0.443	1.066	
**GERD**						
Yes	-0.517	0.645		-1.162	0.641	

Asterisks denote statistical significance from multivariable linear regression on health-related quality of life among adults with osteoarthritis, ***P <0.001; **0.001 ≤ P <0.01; *0.01 ≤ P <.05
>: Greater than; <: Less than
COPD: Chronic obstructive pulmonary disease; GERD: Gastroesophageal reflux disease; Rx: Medication; Ref: reference group; SE: Standard Error; Sig: Significance.

4. Discussion

The findings of this study support that comorbid depression and anxiety are linked to poor HRQoL in adults with OA. Our study filled in some gaps and uncertainties about the relationship between comorbid depression and anxiety and HRQoL domains by using a nationally representative sample of US adults with OA. In our study, we compared four groups of adults with OA in terms of their quality of life: 1) those with OA only, 2) those with OA and depression, 3) those with OA and anxiety, and 4) those with OA and both depression and anxiety. By comparing these groups, we observed that those with anxiety and depression had

worse HRQoL than adults with any of these comorbidities only, or without any of them. Adults with OA and both depression and anxiety have a lower PCS score and MCS score compared to those with OA only (PCS score of 36.84 vs. 43.12, MCS score of 37.47 vs. 51.20), presence of anxiety and depression led to a nearly 6-point reduction in PCS score and a 14-point reduction in MCS score among adults with OA. In our adjusted analysis, the β-coefficient for those with both depression and anxiety (MCS: $\beta = -10.317$) indicates that having anxiety and depression comorbidity, as opposed to no mental comorbidity in adults with OA is associated with a 10-unit reduction in HRQoL score in the MCS domain. These findings indicate the incremental effect of having anxiety and depression on health status and reflect a greater disease burden of these mental illnesses. Our analysis's findings concur with those of other previously published research. For example, a study using data from the Korea National Health and Nutrition Examination Survey revealed lower HRQoL in OA adults with psychiatric disease [17]. As nearly one in ten adults with OA in our study have depression or anxiety, these findings suggest the need for OA point-of-care anxiety and depression screening [34], since they are consistently underdiagnosed and neglected. Many general practitioners and primary care providers often overlook these comorbidities because they either only address the physical aspects of OA or neglect to evaluate their patients' psychological well-being entirely [35]. Understanding these comorbidities is crucial because they can alter OA management and progression. The National Institute for Health and Care Excellence (NICE) has provided guidelines for practitioners to guide them in the holistic evaluation of patients with OA [36]. Therefore, policies and initiatives must promote and support coordinated care that covers OA and mental health management to improve their quality of life, reduce the disease burden, and fill the mental health care gap [37].

Additionally, the results of this study demonstrated that other risk factors including income, education levels, and coexisting hypertension, have an unneglectable impact on HRQoL among adults with OA. In fact, in our descriptive analysis we found that those with high school education level, low income, and those with chronic illnesses have a higher prevalence of depression and anxiety. Previous studies that involved adults in general and adults with OA reported that these are important determinants of HRQoL [6, 18, 27-33]. The negative association between having high school education and physical health (PCS domain of HRQoL), compared to less than a high school education, may be explained by the higher prevalence of depression and anxiety comorbidity from bivariate analysis in this group, as mental health conditions can exacerbate physical health issues through poor health behaviors. A recently published data from the Epidemiology of Chronic Diseases cohort study, a 10-year follow-up for 983 hip and knee OA patients reported that multimorbidity was associated with a higher risk of poor physical function and HRQoL trajectories [38]. Besides, in earlier studies, chronic illnesses are linked to mental health and are associated with a reduced HRQoL in their sufferers because of their chronic nature and management [39-41]. Given that enhancing HRQoL is seen as a noteworthy outcome and a predictor of disease management, it emphasizes the need for comprehensive care that addresses OA management and physical and mental comorbidities. Recognizing income, education, and chronic illnesses as essential factors in adults with OA can guide healthcare providers in identifying of OA individuals who may be at higher risk of experiencing poor HRQoL and tailoring interventions accordingly. For example, targeting those with low education and income with additional preventative strategies can help in mitigate the negative impact of such risk factors and prevent or minimize impairment in the quality of life of individuals with OA.

Furthermore, this study found that OA adults who were employed, lived in Northeast and Mid-west regions, perceived their physical health to be excellent, exercise regularly had higher HRQoL. Employment was particularly associated with improved physical and mental well-being, likely due to financial stability, social engagement, and a sense of purpose. Additionally, individuals with higher incomes generally experienced better health outcomes, emphasizing the role of economic security in maintaining a good quality of life. Perceived health played a crucial role in overall well-being, as individuals who rated their physical health as excellent experienced significantly better quality of life. This suggests that self-assessment of health status is a strong predictor of both physical and mental well-being. Additionally, regional differences in HRQoL were observed, with those residing in the Northeast and Midwest regions reporting better health outcomes. This could be attributed to variations in healthcare access, socioeconomic conditions, environmental factors, or community support systems available in these areas. Physical activity also emerged as a key contributor to improved HRQoL. Prior research investigated the effects of physical activity on OA patients' physical function and HRQoL using a statewide population-based cohort reported that regular physical activity was related to enhanced physical function and HRQoL among individuals with hip and/or Knee OA [13, 42]. The results of this study further highlighted the necessity of managing OA through a multidisciplinary approach that includes lifestyle, psychological, and pharmacological interventions customized to each patient's needs to lessen the negative effects of coexisting mental conditions and, therefore, improve the overall quality of life.

4.1. Study Strengths and Limitations

This study used a large sample size and a nationally representative sample of US adults with OA, which allowed us to obtain a clinically insightful estimate of the relationship between depression

and anxiety and HRQoL in adults with OA. However, this study has some limitations. First, the cross-sectional design of our study limits its ability to determine the causal relationship between depression, anxiety, and HRQoL. Second, specific critical confounders were not available on MEPS data and have not been adjusted for in the analysis, including osteoarthritis-related variables (i.e., treatment, the severity of their illness, disease subtypes, OA-related pain, pain-severity, and pain location (hip vs. knee joint)) which can interfere with a person's ability to function normally and have a negative relationship with HRQoL [17, 38, 43, 44]. These essential confounders were overlooked, which all have a significant role in influencing HRQoL in adults with OA and are crucial to comprehending the intricate relationship between mental health and OA patients' HRQoL. Third, missing these critical OA-related variables in the analysis may have introduced a potential omitted variable bias and weakened the study's findings and conclusion; thus, the results of this study may have overestimated the direct impact of anxiety and depression on HRQoL in persons with osteoarthritis, therefore, adding these crucial factors should be the top priority for future research. However, in this study's analysis compared to previously published research, an adjustment for several essential confounders that can potentially affect the HRQoL, such as social determinants, chronic illnesses, physical activity, health insurance coverage, and perceived health, were included to produce greater confidence in detecting the actual effect of mental health on HRQoL in adults with OA [27-33]. Finally, the fact that data on HRQoL and OA symptoms are mostly self-reported introduces a potential source for biases such as underreporting or misreporting bias.

4.2. Clinical Practice, Policy and Public Health Implications

The clinical practice implication of the results of our research should reassure us that physicians, rheumatologists, and mental health professionals should incorporate routine screening for

anxiety and depression in adults with OA, especially when patients or their families report symptoms [45]. This can help minimize suffering, improve symptom management, and enhance HRQoL. Clinicians should adhere to the NICE guidelines on identifying and treating depression, utilizing a combination of pharmacotherapy, cognitive-behavioral therapy (CBT), physical therapies, and structured activities to optimize treatment outcomes [46]. Some research suggested that the use of CBT, education programs, pharmacotherapy, and physical exercise are linked to the short-term reduction of depressed symptoms in older people with OA [35, 47]. Besides, healthcare providers should adopt an integrated pain management approach, combining pharmacologic treatments (e.g., simple analgesics, NSAIDs, COX-2 inhibitors) with non-pharmacologic interventions (e.g., movement meditation, acupuncture, and psychological therapy) to reduce pain-related anxiety and depression in OA patients [48-50].

The policy implications of this study suggest that policymakers should develop and enforce guidelines that promote the integration of mental health screening into routine OA care at primary and specialty healthcare settings [34]. Governments and health insurance providers must enhance access to affordable mental health services by reducing financial barriers for uninsured and underinsured populations. This could include expanding publicly funded mental health programs and increasing coverage for psychological services in health insurance plans. This is especially important due to the high cost of mental health care, as people with mental illnesses have historically faced major barriers to seeking mental health care for the uninsured and even those who are insured [51].

The public health implications encourage lifestyle changes like physical activity. Physical activity equivalent to 150 minutes/week of moderate-intensity exercise or at least 45 minutes/week of moderate-intensity can have a detrimental positive impact on HRQoL [42, 52,

53], decreased OA pain [53], improve physical function of OA patients [52, 53]. Public health agencies should leverage exercise-based interventions as a cost-effective alternative to antidepressant treatment, given its comparable benefits in reducing depression and anxiety in OA patients [54, 55].

5. Conclusions

The relationships between anxiety, depression, and a low HRQoL in patients with OA was found in this study. This study also revealed the negative effect of low education and low income on HRQoL while highlighting the advantages of employment and regular exercise. It is crucial for future research to thoroughly evaluate patient-centered clinical approaches and care and management policies. This understanding of risk factors that hinder patients with OA, which increase anxiety and depression risk and therefore compromise the HRQoL in individuals with OA, is worthy.

List of Abbreviations

HRQoL Health-Related Quality of Life, MCS mental health component score, PCS physical health component score.

Declarations

Funding

Authors' Contributions

MA: design of the study, searching the literature, manuscript writing, and analyzing the findings; TA: searching the literature, and manuscript writing. Both authors read and approved the final manuscript.

Institutional Review Board Statement

No ethical review or approval was needed as we have utilized a publicly accessible, the Medical Expenditure Panel Survey (MEPS) database.

Informed Consent Statement

Medical Expenditure Panel Survey (MEPS) database is publicly accessible secondary data, therefore, patient consent was waived for this research.

Data Availability Statement

The dataset used in this study is available from the MEPS database at this URL: https://meps.ahrq.gov/data_stats.

Conflicts of Interest

The author affirms that there are no conflicts of interest related to the publication of this paper

References

1. Leifer VP, Katz JN, Losina E. The burden of OA-health services and economics. Osteoarthritis and Cartilage. 2022;30(1):10-6.

2. Steinmetz JD, Culbreth GT, Haile LM, Rafferty Q, Lo J, Fukutaki KG, et al. Global, regional, and national burden of osteoarthritis, 1990–2020 and projections to 2050: a systematic analysis for the Global Burden of Disease Study 2021. The Lancet Rheumatology. 2023;5(9):e508-e22.

3. Allen KD, Thoma LM, Golightly YM. Epidemiology of osteoarthritis. Osteoarthritis and Cartilage. 2022;30(2):184-95.

4. Scheuing WJ, Reginato AM, Deeb M, Acer Kasman S. The burden of osteoarthritis: Is it a rising problem? Best Practice & Research Clinical Rheumatology. 2023;37(2):101836.

5. Rothrauff B, Tang Q, Wang J, He J. Osteoarthritis is positively associated with self-reported sleep trouble in older adults. Aging Clinical and Experimental Research. 2022;34(11):2835-43.

6. Chang W-Y, Choi S, Yoo SJ, Lee J, Lim C. Factors Associated with Osteoarthritis and Their Influence on Health-Related Quality of Life in Older Adults with Osteoarthritis: A Study Based on the 2020 Korea National Health and Nutrition Examination Survey. International Journal of Environmental Research and Public Health. 2023;20(12):6073.

7. Zhao X, Shah D, Gandhi K, Wei W, Dwibedi N, Webster L, et al. Clinical, humanistic, and economic burden of osteoarthritis among noninstitutionalized adults in the United States. Osteoarthritis and Cartilage. 2019;27(11):1618-26.

8. Savvari P, Skiadas I, Papadakis SA, Psychogios V, Argyropoulou OD, Pastroudis AP, et al. The impact of moderate to severe osteoarthritis on the physical performance and quality of

life: a cross-sectional study in Greek patients (PONOS study). BMC Musculoskeletal Disorders. 2023;24(1):651.

9. Jin X, Liang W, Zhang L, Cao S, Yang L, Xie F. Economic and humanistic burden of osteoarthritis: an updated systematic review of large sample studies. Pharmacoeconomics. 2023;41(11):1453-67.

10. Yan H, Guo J, Zhou W, Dong C, Liu J. Health-related quality of life in osteoarthritis patients: a systematic review and meta-analysis. Psychology, Health & Medicine. 2022;27(8):1859-74.

11. Wang ST, Ni GX. Depression in Osteoarthritis: Current Understanding. Neuropsychiatr Dis Treat. 2022;18:375-89.

12. Sambamoorthi U, Shah D, Zhao X. Healthcare burden of depression in adults with arthritis. Expert Rev Pharmacoecon Outcomes Res. 2017;17(1):53-65.

13. Fonseca-Rodrigues D, Rodrigues A, Martins T, Pinto J, Amorim D, Almeida A, et al. Correlation between pain severity and levels of anxiety and depression in osteoarthritis patients: a systematic review and meta-analysis. Rheumatology. 2021;61(1):53-75.

14. Rosemann T, Gensichen J, Sauer N, Laux G, Szecsenyi J. The impact of concomitant depression on quality of life and health service utilisation in patients with osteoarthritis. Rheumatology International. 2007;27(9):859-63.

15. Tsuji T, Nakata K, Vietri J, Jaffe DH. The added burden of depression in patients with osteoarthritis in Japan. ClinicoEconomics and Outcomes Research. 2019;11:411-21.

16. Agarwal P, Sambamoorthi U. Healthcare Expenditures Associated with Depression Among Individuals with Osteoarthritis: Post-Regression Linear Decomposition Approach. Journal of General Internal Medicine. 2015;30(12):1803-11.

17. Lee Y, Lee S-H, Lim SM, Baek SH, Ha I-H. Mental health and quality of life of patients with osteoarthritis pain: The sixth Korea National Health and Nutrition Examination Survey (2013–2015). PLOS ONE. 2020;15(11):e0242077.

18. Kim DJ. A Study on the Physical Activities, Mental Health, and Health-Related Quality of Life of Osteoarthritis Patients. Osong Public Health Res Perspect. 2019;10(6):368-75.

19. Karimi M, Brazier J. Health, Health-Related Quality of Life, and Quality of Life: What is the Difference? PharmacoEconomics. 2016;34(7):645-9.

20. Pinto Barbosa S, Marques L, Sugawara A, Toledo F, Imamura M, Battistella L, et al. Predictors of the Health-Related Quality of Life (HRQOL) in SF-36 in Knee Osteoarthritis Patients: A Multimodal Model With Moderators and Mediators. Cureus. 2022;14(7):e27339.

21. Tavares DRB, Moça Trevisani VF, Frazao Okazaki JE, Valéria de Andrade Santana M, Pereira Nunes Pinto AC, Tutiya KK, et al. Risk factors of pain, physical function, and health-related quality of life in elderly people with knee osteoarthritis: A cross-sectional study. Heliyon. 2020;6(12):e05723.

22. Agency for Healthcare Research and Quality. Medical Expenditure Panel Survey HC-231: 2021 Medical Conditions File. [cited 2024]; Available from: https://meps.ahrq.gov/data_stats/download_data/pufs/h231/h231doc.pdf.

23. Maslak JP, Jenkins TJ, Weiner JA, Kannan AS, Patoli DM, McCarthy MH, et al. Burden of Sciatica on US Medicare Recipients. JAAOS - Journal of the American Academy of Orthopaedic Surgeons. 2020;28(10):e433-e9.

24. Selim AJ, Rothendler JA, Qian SX, Bailey HM, Kazis LE. The History and Applications of the Veterans RAND 12-Item Health Survey (VR-12). The Journal of Ambulatory Care Management. 2022;45(3):161-70.

25. Kazis LE, Rogers WH, Rothendler J, Qian S, Selim A, Edelen MO, et al. Outcome Performance Measure Development for Persons with Multiple Chronic Conditions. Santa Monica, CA: RAND Corporation; 2017.

26. Cheak-Zamora NC, Wyrwich KW, McBride TD. Reliability and validity of the SF-12v2 in the medical expenditure panel survey. Quality of Life Research. 2009;18(6):727-35.

27. Heyworth ITM, Hazell ML, Linehan MF, Frank TL. How do common chronic conditions affect health-related quality of life? British Journal of General Practice. 2009;59(568):e353-8.

28. Keles H, Ekici A, Ekici M, Bulcun E, Altinkaya V. Effect of chronic diseases and associated psychological distress on health-related quality of life. Internal Medicine Journal. 2007;37(1):6-11.

29. Kivits J, Erpelding ML, Guillemin F. Social determinants of health-related quality of life. Rev Epidemiol Sante Publique. 2013;61 Suppl 3:S189-94.

30. Saffari M, Meybodi MKE, Ghanizadeh G, Koenig HG. Determinants of health status among patients with knee or hip osteoarthritis: the role of demographic, clinical and health related quality of life variables. Journal of Musculoskeletal Research. 2014;17(01):1450004.

31. Yuniarwati W, Subijanto AA, Prasetyo H. Determinants of Quality of Life among Patient with Knee Osteoarthritis. Journal of Epidemiology and Public Health. 2019;4(4):320-7.

32. Luong M-LN, Cleveland RJ, Nyrop KA, Callahan LF. Social Determinants and Osteoarthritis Outcomes. Aging Health. 2012;8(4):413-37.

33. Bharmal M, Thomas J, 3rd. Health insurance coverage and health-related quality of life: analysis of 2000 Medical Expenditure Panel Survey data. J Health Care Poor Underserved. 2005;16(4):643-54.

34. Mallen CD, Nicholl BI, Lewis M, Bartlam B, Green D, Jowett S, et al. The effects of implementing a point-of-care electronic template to prompt routine anxiety and depression screening in patients consulting for osteoarthritis (the Primary Care Osteoarthritis Trial): A cluster randomised trial in primary care. PLoS Med. 2017;14(4):e1002273.

35. Sharma A, Kudesia P, Shi Q, Gandhi R. Anxiety and depression in patients with osteoarthritis: impact and management challenges. Open Access Rheumatology: Research and Reviews. 2016;8:103-13.

36. National Institute for Health and Care Excellence. Osteoarthritis in over 16s: diagnosis and management. 2022; Available from: https://www.nice.org.uk/guidance/ng226/resources/osteoarthritis-in-over-16s-diagnosis-and-management-pdf-66143839026373.

37. Gleicher Y, Croxford R, Hochman J, Hawker G. A prospective study of mental health care for comorbid depressed mood in older adults with painful osteoarthritis. BMC Psychiatry. 2011;11:147.

38. Costa D, Lopes DG, Cruz EB, Henriques AR, Branco J, Canhão H, et al. Trajectories of physical function and quality of life in people with osteoarthritis: results from a 10-year population-based cohort. BMC Public Health. 2023;23(1):1407.

39. Park Y, Park K. Health-related quality of life and depressive symptoms of patients with chronic diseases and the general population before and during the COVID-19 pandemic in Korea. Front Psychol. 2023;14:1117369.

40. Maynié-François C, Burtey S. Comparing health-related quality of life in chronic diseases: the importance of analyzing references. Nature Reviews Disease Primers. 2019;5(1): e27.

41. Megari K. Quality of Life in Chronic Disease Patients. Health Psychol Res. 2013;1(3):24.

42. Anokye NK, Trueman P, Green C, Pavey TG, Taylor RS. Physical activity and health related quality of life. BMC Public Health. 2012;12:624.

43. Chang J, Yuan Y, Fu M, Wang D. Health-related quality of life among patients with knee osteoarthritis in Guangzhou, China: a multicenter cross-sectional study. Health and Quality of Life Outcomes. 2023;21(1):50.

44. Montero A, Mulero J-F, Tornero C, Guitart J, Serrano M. Pain, disability and health-related quality of life in osteoarthritis—joint matters: an observational, multi-specialty trans-national follow-up study. Clinical Rheumatology. 2016;35(9):2293-305.

45. Frankish H, Boyce N, Horton R. Mental health for all: a global goal. The Lancet. 2018;392(10157):1493-4.

46. National Institute for Health and Care Excellence. Depression in adults: treatment and management. 2022; Available from: https://www.nice.org.uk/guidance/ng222.

47. Yohannes AM, Caton S. Management of depression in older people with osteoarthritis: A systematic review. Aging & Mental Health. 2010;14(6):637-51.

48. Burley CV, Casey A-N, Jones MD, Wright KE, Parmenter BJ. Nonpharmacological approaches for pain and symptoms of depression in people with osteoarthritis: systematic review and meta-analyses. Scientific Reports. 2023;13(1):15449.

49. Manyanga T, Froese M, Zarychanski R, Abou-Setta A, Friesen C, Tennenhouse M, et al. Pain management with acupuncture in osteoarthritis: a systematic review and meta-analysis. BMC Complementary and Alternative Medicine. 2014;14:312.

50. Walker-Bone K, Javaid K, Arden N, Cooper C. Medical management of osteoarthritis. BMJ. 2000;321(7266):936-40.

51. Rowan K, McAlpine DD, Blewett LA. Access and cost barriers to mental health care, by insurance status, 1999-2010. Health Aff. 2013;32(10):1723-30.
52. Lopes DG, Costa D, Cruz EB, Mendonça N, Henriques AR, Branco J, et al. Association of physical activity with physical function and quality of life in people with hip and knee osteoarthritis: longitudinal analysis of a population-based cohort. Arthritis Research & Therapy. 2023;25(1):14.
53. Kraus VB, Sprow K, Powell KE, Buchner D, Bloodgood B, Piercy K, et al. Effects of Physical Activity in Knee and Hip Osteoarthritis: A Systematic Umbrella Review. Med Sci Sports Exerc. 2019;51(6):1324-39.
54. Dinas PC, Koutedakis Y, Flouris AD. Effects of exercise and physical activity on depression. Irish Journal of Medical Science. 2011;180(2):319-25.
55. Kandola A, Ashdown-Franks G, Hendrikse J, Sabiston CM, Stubbs B. Physical activity and depression: Towards understanding the antidepressant mechanisms of physical activity. Neuroscience & Biobehavioral Reviews. 2019;107:525-39.

Manuscript ID: PONE-D-24-50708
Mental Health and Health-Related Quality of Life among Adults with Osteoarthritis: A National Population-Based Study

Response to Reviewer Comments

We appreciate the reviewer's thoughtful comments and suggestions, which have helped improve the clarity and quality of our manuscript. Below is our detailed response to each comment:

Data Analysis:

Comment 1: *Inclusion of Candidate Variables in Multivariable Linear Regression Model*

> **Response:** Thank you for this comment; we have now clarified the selection process of variables in the Methods section. Variables were included based on their theoretical relevance, clinical importance, and prior literature supporting their association with health-related quality of life (HRQoL) in osteoarthritis (OA) patients. Additionally, a bivariate analysis was performed, and only variables with a p-value <0.05 were considered for the multivariable regression model.

Results:

Comment 2: *Likelihood Ratio p-value Indication*

> **Response:** We have ensured that the reported p-values are based on the likelihood ratio test where appropriate and have explicitly indicated this in the text and tables.

Comment 3: *Reporting All Significant Results*

> **Response:** We have revised the results section to ensure that all significant findings are reported.

Comment 4: *Specificity about Domains Being Reported*

> **Response:** Thanks for pointing out, now we have specify whether the reported HRQoL results pertain to the Physical Component Summary (PCS) or the Mental Component Summary (MCS) to enhance clarity.

Comment 5: *Comparison with the Reference Group*

> **Response:** Thanks for this comment; we have ensured that all the reported regression results include a clear comparison with the reference group.

Manuscript ID: PONE-D-24-50708
Mental Health and Health-Related Quality of Life among Adults with Osteoarthritis: A National Population-Based Study

Discussion:

Comment 6: *Addressing Significant vs. Insignificant Results*

> **Response:** The discussion has been revised to emphasize significant results while removing discussion on insignificant findings (i.e., gender was previously discussed despite being statistically insignificant; this has now been removed).

Comment 7: *Comprehensive Reporting of Key Variables*

> **Response:** As per the reviewer recommendations, we have expanded the discussion to cover all significant factors, including perceived health, employment, region, and income, as they were significantly associated with HRQoL. We have also clarified the role of education by providing possible explanations for the observed findings and comparing them with previous studies.

Recommendations:

Comment 8: *Specificity in Recommendations*

> **Response:** Thanks for the suggestion. Recommendations have been revised to clearly address specific entities such as healthcare providers, policymakers, and public health organizations.

Comment 9: *Re-editing and Completing References*

> **Response:** The reference section has been thoroughly revised to ensure completeness, correct formatting, and accuracy.
