# Supplementary material for: Mental health and health-related quality of life among adults with osteoarthritis: A national population-based study
Source: PLoS One. 2025 Jun 18;20(6):e0325780. doi: 10.1371/journal.pone.0325780 (PMC12176145; doi:10.1371/journal.pone.0325780)
Supplement: Attachment — Submitted filename: OA_HRQoL_Response to reviewer_05-03-2025.docx [file pone.0325780.s002.docx]

**Response to Reviewer Comments**

We appreciate the reviewer’s thoughtful comments and suggestions, which have helped improve the clarity and quality of our manuscript. Below is our detailed response to each comment:

**Data Analysis:**

**Comment 1: *Inclusion of Candidate Variables in Multivariable Linear Regression Model***

**Response:** Thank you for this comment; we have now clarified the selection process of variables in the Methods section. Variables were included based on their theoretical relevance, clinical importance, and prior literature supporting their association with health-related quality of life (HRQoL) in osteoarthritis (OA) patients. Additionally, a bivariate analysis was performed, and only variables with a p-value <0.05 were considered for the multivariable regression model.

**Results:**

**Comment 2:** ***Likelihood Ratio p-value Indication***

**Response:** We have ensured that the reported p-values are based on the likelihood ratio test where appropriate and have explicitly indicated this in the text and tables.

**Comment 3:** ***Reporting All Significant Results***

**Response:** We have revised the results section to ensure that all significant findings are reported.

**Comment 4:** ***Specificity about Domains Being Reported***

**Response:** Thanks for pointing out, now we have specify whether the reported HRQoL results pertain to the Physical Component Summary (PCS) or the Mental Component Summary (MCS) to enhance clarity.

**Comment 5:** ***Comparison with the Reference Group***

**Response:** Thanks for this comment; we have ensured that all the reported regression results include a clear comparison with the reference group.

**Discussion:**

**Comment 6:** ***Addressing Significant vs. Insignificant Results***

**Response:** The discussion has been revised to emphasize significant results while removing discussion on insignificant findings (i.e., gender was previously discussed despite being statistically insignificant; this has now been removed).

**Comment 7:** ***Comprehensive Reporting of Key Variables***

**Response:** As per the reviewer recommendations, we have expanded the discussion to cover all significant factors, including perceived health, employment, region, and income, as they were significantly associated with HRQoL. We have also clarified the role of education by providing possible explanations for the observed findings and comparing them with previous studies.

**Recommendations:**

**Comment 8:** ***Specificity in Recommendations***

**Response:** Thanks for the suggestion. Recommendations have been revised to clearly address specific entities such as healthcare providers, policymakers, and public health organizations.

**Comment 9:** ***Re-editing and Completing References***

**Response:** The reference section has been thoroughly revised to ensure completeness, correct formatting, and accuracy.
